# Plekhm2 acts as an autophagy modulator in murine heart and cardiofibroblasts

**DOI:** 10.1038/s41598-024-65670-5

**Published:** 2024-06-28

**Authors:** Sharon Etzion, Raneen Hijaze, Liad Segal, Sofia Pilcha, Dana Masil, Or Levi, Sigal Elyagon, Aviva Levitas, Yoram Etzion, Ruti Parvari

**Affiliations:** 1 Regenerative Medicine and Stem Cell (RMSC) Research Center, Ben-Gurion University of the Negev, P.O. Box 653, 84105 Be’er-Sheva, Israel; 2 Department of Microbiology, Immunology and Genetics, Faculty of Health Sciences, Ben-Gurion University of the Negev, 84101 Be’er-Sheva, Israel; 3 Department of Physiology and Cell Biology, Faculty of Health Sciences, Ben-Gurion University of the Negev, 84101 Be’er-Sheva, Israel; 4 Department of Pediatric Cardiology, Soroka University Medical Center, Ben-Gurion University of the Negev, 84101 Be’er-Sheva, Israel; 5 National Institute for Biotechnology, Ben-Gurion University of the Negev, 84101 Be’er-Sheva, Israel

**Keywords:** Cardiology, Cardiovascular biology

## Abstract

Plekhm2 is a protein regulating endosomal trafficking and lysosomal distribution. We recently linked a recessive inherited mutation in *PLEKHM2* to a familial form of dilated cardiomyopathy and left ventricular non-compaction. These patients’ primary fibroblasts exhibited abnormal lysosomal distribution and autophagy impairment. We therefore hypothesized that loss of PLEKHM2 impairs cardiac function via autophagy derangement. Here, we characterized the roles of Plekhm2 in the heart using global *Plekhm2* knockout (PLK2-KO) mice and cultured cardiac cells. Compared to littermate controls (WT), young PLK2-KO mice exhibited no difference in heart function or autophagy markers but demonstrated higher basal AKT phosphorylation. Older PLK2-KO mice had body and heart growth retardation and increased LC3II protein levels. PLK2-KO mice were more vulnerable to fasting and, interestingly, impaired autophagy was noted in vitro, in Plekhm2-deficient cardiofibroblasts but not in cardiomyocytes. PLK2-KO hearts appeared to be less sensitive to pathological hypertrophy induced by angiotensin-II compared to WT. Our findings suggest a role of Plekhm2 in murine cardiac autophagy. Plekhm2 deficiency impaired autophagy in cardiofibroblasts, but the autophagy in cardiomyocytes is not critically dependent on Plekhm2. The absence of Plekhm2 in mice appears to promote compensatory mechanism(s) enabling the heart to manage angiotensin-II-induced stress without detrimental consequences.

## Introduction

Macroautophagy (hereafter referred to as autophagy) is a highly regulated and conserved cellular process that involves lysosome-mediated degradation of unnecessary or damaged intracellular components^1,2^. Autophagy allows an orderly degradation and recycling of cellular components by promoting the isolation of proteins, organelles, and part of the cytoplasm within a transient double membrane that expands and closes to become an autophagosome. The autophagosome fuses with the lysosome to form the autolysosome and promote cargo degradation^3^. Therefore, autophagy allows cells to maintain homeostasis and survive various stress conditions.

Autophagy plays a major role in the heart. It preserves cardiac structure and function under normal conditions and is activated during stress to limit damage to cardiac cells^3^. Moreover, autophagy is required for normal cardiac development^4^. The autophagy level in the heart is altered in response to stresses such as ischemia/reperfusion^5^ and nutrient deprivation^4^ but is also triggered by chronic conditions such as cardiac hypertrophy and heart failure^4,5^. Cardiac remodeling involves an increase in cardiomyocyte cell death via apoptosis, necrosis, and autophagy^5^. Impaired autophagy contributes to the development of cardiac proteinopathy and doxorubicin-induced cardiomyopathy and is involved in the development of diabetes and aging-induced cardiac abnormalities^6,7^. Massive activation of autophagy is also detrimental for the heart in certain stress conditions, such as reperfusion injury^3,5^.

Dilated cardiomyopathy (DCM) is a cause of substantial morbidity and mortality. It is associated with dilation and impaired contraction of the left or both ventricles and is a major cause of heart failure worldwide^8^. Familial DCM is usually transmitted with autosomal dominant inheritance, although recessive and X-linked inheritance patterns have been described as well^8–11^. Alterations in myocardial autophagy have been observed in the context of DCM^5,12,13^. However, it is unclear whether autophagy plays a cardioprotective role or is part of the tissue damage in this context.

In a previous report^9^, we described a recessively inherited form of DCM and left ventricular (LV) non-compaction (LVNC) presenting with severe cardiac dysfunction in young adulthood. The only putative causative mutation was found in the pleckstrin homology domain-containing family M member 2 (*PLEKHM2*) gene, and we thus linked the mutation to the disease. Plekhm2, also known as SifA and kinesin-interacting protein (Skip), interacts with kinesin-1, affecting endosomal trafficking. In conjunction with the lysosomal GTPase Arl8 and BORC complex, Plekhm2 is required for normal lysosomal distribution^14,15^. Accordingly, in that report, we showed that the patient’s primary fibroblasts exhibited an abnormal subcellular distribution of endosomes and lysosomes and impaired autophagy^9^. Recently, Atkins et al.^16^ reported a 21-year-old woman with DCM and prominent LV apical trabeculations associated with compound heterozygous loss-of-function variations in the *PLEKHM2* gene. They demonstrated an absence of PLEKHM2 protein from the myocardial tissue, supporting our identified association between PLEKHM2 dysfunction and DCM^9^. More recently, a study of cardiomyocytes derived from human induced pluripotent stem cells with PLEKHM2 loss of function also found dysregulated autophagy as well as reduced contractility and impaired calcium handling^17^.

In the present study, we aimed to investigated the roles played by Plekhm2 in the murine heart both in regard to autophagy regulation and in terms of cardiac morphology and function under basal conditions and in the presence of neurohormonal stress mimicked by Angiotensin-II (AngII) infusion. Overall, our findings demonstrate a rather complex involvement of Plekhm2 in cardiac biology. We show that 3-month-old *Plekhm2* knockout (PLK2-KO) mice have normal heart function and a balanced autophagy process while 12-month-old PLK2-KO mice show body and heart growth retardation. Elevated levels of LC3II and p62 proteins were observed in the PLK2-KO mice, suggesting an accumulation of autophagosomes. PLK2-KO mice were also more vulnerable to nutrient deprivation than littermate controls but were surprisingly, less sensitive to pathological hypertrophy induced by AngII. Our findings further indicate that basal AKT phosphorylation is higher in young PLK2-KO mice, which possibly serves as a compensatory mechanism. In vitro, we found that deletion of murine *Plekhm2* leads to impaired autophagy in murine cardiofibroblasts but not in the cardiomyocytes.

## Results

**Figure 1:**
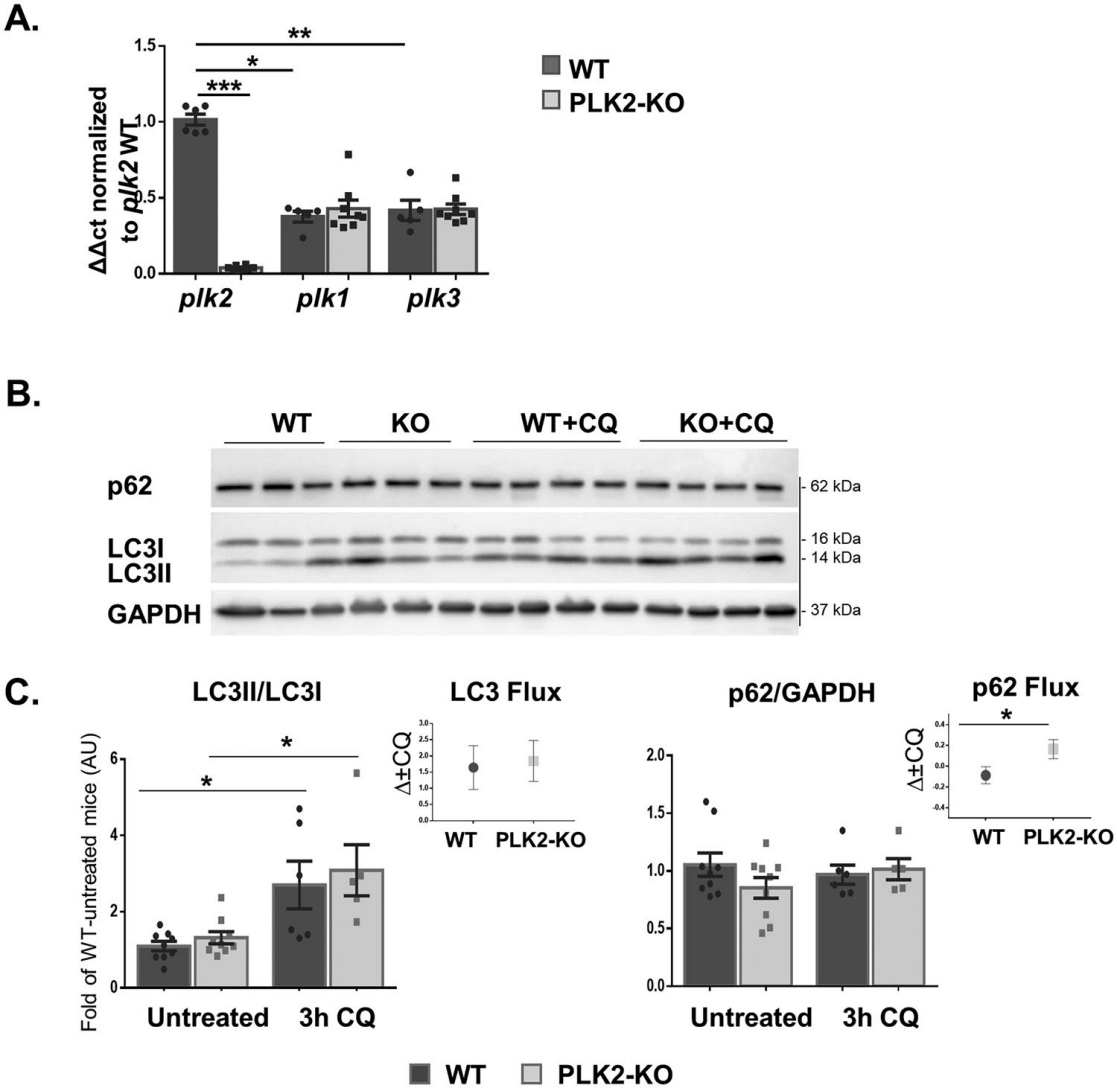
Quantitation of Plekhm mRNA levels and basic autophagy in PLK2-KO mice. (**A**) Levels of *plk2*, *plk1*, and *plk3* mRNA were examined by RT real-time quantitative PCR (qPCR) in hearts from 3-month-old female and male mice with global Plekhm2 knockout (PLK2-KO, n = 8) and their normal siblings (WT, n = 5–6). The results are presented as the fold of *plk2* expression in WT mice. GAPDH (*gpdh*) served as an internal control. (**B**) Representative example of WB results for LC3I, LC3II, p62, and GAPDH protein levels in 3-month-old female mice subjected to 3 h of 80 mg/kg CQ. (**C**) Quantitative analysis of the LC3II/LC3I ratio and p62/GAPDH. Autophagy flux was calculated as the delta between CQ-injected mice and untreated mice in each group (inset). *p < 0.05, **p < 0.01, and ***p < 0.001 by Kruskal–Wallis test with Dunn’s multiple comparison post-test and the Mann–Whitney nonparametric test.

To uncover the roles of Plekhm2 in the heart, we examined the phenotype of adult mice that were homozygous for the Plekhm2tm1a cassette, which knocked out the *Plekhm2* gene in a global manner (PLK2-KO). Plekhm2 protein was difficult to detect by western blot (WB) assay in heart extracts. However, we were able to confirm a marked reduction in *Plekhm2* (*plk2*) mRNA by RT real-time quantitative PCR (qPCR) in both male and female PLK2-KO mice compared to their siblings homozygous for the normal allele (WT), presumably due to nonsense-mediated mRNA decay (Fig. 1A). In addition, the cardiac *Plekhm2* mRNA level of WT mice was found to be higher than that of its homologs *Plekhm1* (*plk1*) and *Plekhm3* (*plk3*), which were recently reported to preserve cardiac function under stress conditions^18,19^. Neither of these two genes mRNA levels were increased in the PLK2-KO hearts (Fig. 1A), suggesting that the homologs do not compensate for the absence of Plekhm2 in our model.

### Plekhm2 KO mice demonstrate imbalanced autophagy with aging

Cardiac autophagy and autophagy flux were examined in the cardiac lysates of 3-month-old female mice 3 h after intraperitoneal injection of chloroquine (CQ, 80 mg/kg), an autophagy inhibitor that mainly acts by impairing autophagosome-lysosome fusion, thereby leading to autophagosome accumulation^20,21^ and affecting cardiac function^22^. LC3I, LC3II, and p62 protein levels and the LC3II/LC3I ratio were examined as indicators of the autophagy process. Increase in LC3II and p62 protein levels and the LC3II/LC3I ratio can reflect the accumulation of autophagosomes^3^. Indeed, CQ increased the LC3II/LC3I ratio in both WT and PLK2-KO mice (*p* = 0.02 and 0.03, respectively). However, we detected no difference between CQ-treated PLK2-KO mice and their control siblings (WT). In addition, no noticeable change in the p62 protein level was detected in response to CQ (Fig. 1B, C). Calculation of the autophagy flux (the delta between CQ-treated and untreated results, inset) also indicated no difference in the LC3II/LC3I ratio but demonstrated a slight but significant (*p* = 0.04) increase in the p62 flux in PLK2-KO mice.

**Figure 2:**
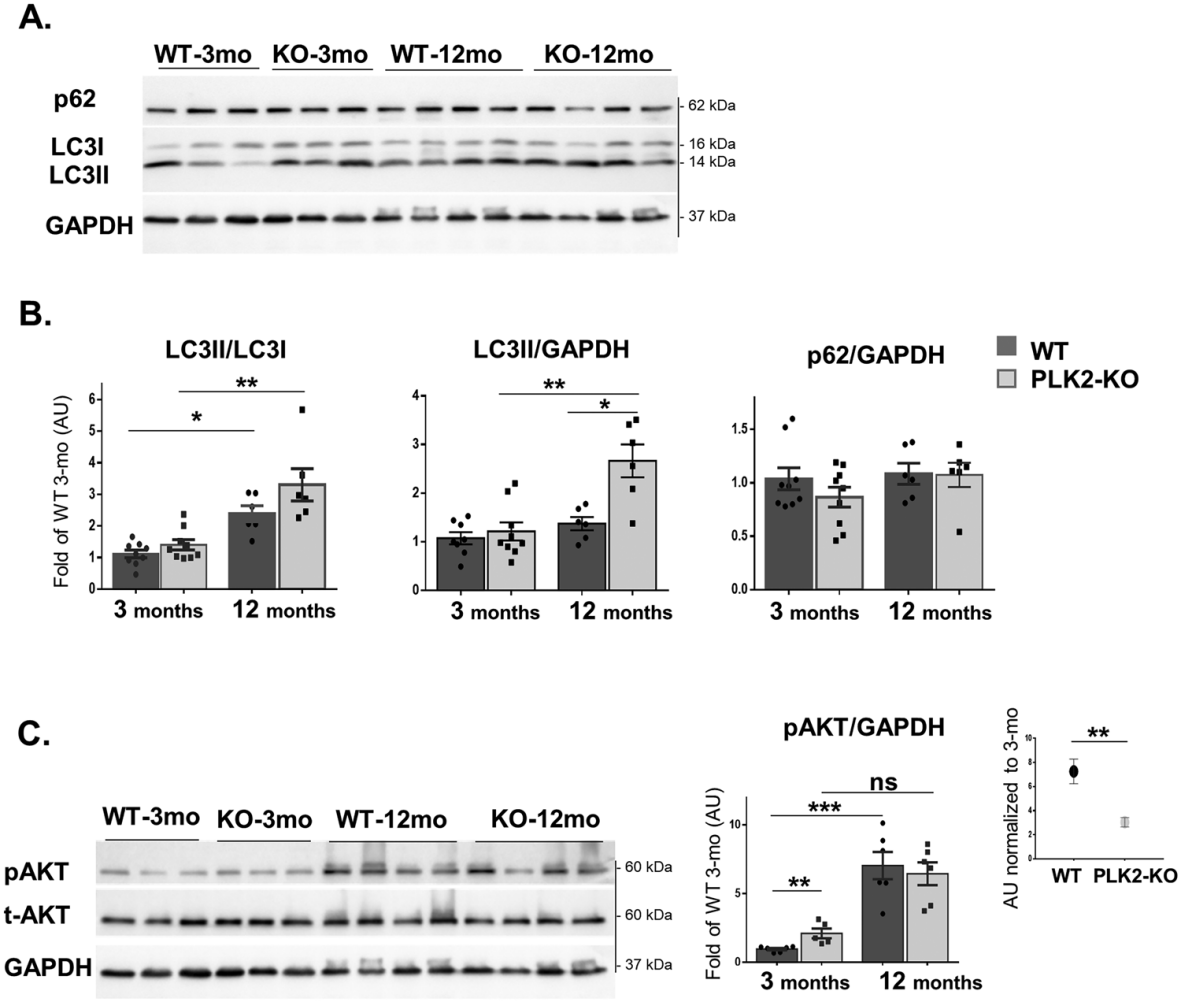
Impaired autophagy signaling in aged PLK2-KO female mice and changes in phosphorylated AKT^473^. (**A**) Representative example of WB results for LC3I, LC3II, p62, and GAPDH from heart lysates. (**B**) Quantitation of protein expression levels in aged mice (12 months old) in comparison to 3-month-old mice: LC3II/LC3I ratio (left) and normalized expression of LC3II (middle) and p62 (right) to GAPDH. The results are displayed as the fold of 3-month-old WT mice. (**C**). Representative example of WB results for phosphorylated AKT^473^ (pAKT^473^), total AKT (t-AKT), and GAPDH (left) and quantification of pAKT^473^/GAPDH (right). Normalized results of aged mice to related 3-month-old mice accentuate the lower activity of pAKT^473^ in PLK2-KO mice with aging (inset). The results are displayed as mean ± SEM (n = 5–8 mice in each group). Statistical analyses were conducted as described in the Methods. *p < 0.05, **p < 0.01, and ***p < 0.001.

We next examined autophagy markers in 12-month-old (“aged”) female mice. Compared with 3-month-old mice, aged PLK2-KO and WT mice both demonstrated an increase in the LC3II/LC3I ratio (*p* = 0.01 and 0.008, respectively; Fig. 2A, B). Although the LC3II/LC3I ratio was not significantly different between PLK2-KO and WT mice at 12 months of age, the LC3II/GAPDH level was higher in PLK2-KO mice (*p* = 0.026). p62 did not exhibit a difference in the aged PLK2-KO hearts compared with aged WT. These results suggest increased accumulation of autophagosomes in PLK2-KO mice with aging (see Discussion).

### Plekhm2 KO mice demonstrate growth retardation and minor cardiac dysfunction with aging

The characterization of 3-month-old PLK2-KO mice using gravimetric and echocardiographic measurements did not reveal differences from both female and male WT mice (Supplementary Tables S1 (female) and S2 (male) online). Cardiac function examined in 6- and 12-month-old female mice (Supplementary Tables S1 and S3 online) also indicated no apparent changes between WT and PLK2-KO mice (e.g., ejection fraction [EF]: WT vs. PLK2-KO, 54.3% ± 4.7% vs. 50.4% ± 2.3% at 12 months old; *p* = 0.9). However, the aged PLK2-KO female mice exhibited a reduced stroke volume and cardiac output (Supplementary Fig. S1 and Table S1 online). The increase in body weight and LV mass with age was moderate in PLK2-KO mice compared with their littermate controls (Supplementary Fig. 1S online). These results, which indicate global growth impairment, were even more pronounced in the hearts of PLK2-KO mice, as emphasized by the lower heart weight (HW)/tibia length (TL) ratio of these mice following aging (*p* = 0.009; Supplementary Table S1 online).

AKT is a serine/threonine kinase that is essential for the regulation of cellular functions, including protein synthesis, cell growth, apoptosis, and autophagy. AKT stimulates mTORC1 activity, which represses autophagy^23^. Total AKT (tAKT) was uniformly expressed in both WT and PLK2-KO mice. However, the pAKT^473^ level was higher in 3-month-old PLK2-KO mice than in 3-month-old WT mice (*p* = 0.004; Fig. 2C). With aging, the activity of the AKT pathway was markedly increased in the WT mice (*p* < 0.001) but not in the PLK2-KO mice (Fig. 2C). Notably, the already elevated level of pAKT in the PLK2-KO mice increased to a lesser extent than in the WT, as emphasized by the normalized value of the aged mice relative to the corresponding 3-month-old mice (WT vs. PLK2-KO, 7.2 ± 1.0-fold vs. 3.0 ± 0.4-fold, respectively; *p* = 0.009; Fig. 2C, inset). Thus, it is possible that the baseline activation of AKT signaling in young PLK2-KO mice is compensating for the abnormal accumulation of autophagosomes in an attempt to maintain normal cardiac function. Based on these results, we suggest that the deletion of Plekhm2 promotes an unbalanced autophagy during aging with consequent retardation of cardiac growth.

### Plekhm2 KO mice demonstrate excessive autophagy following starvation

**Figure 3:**
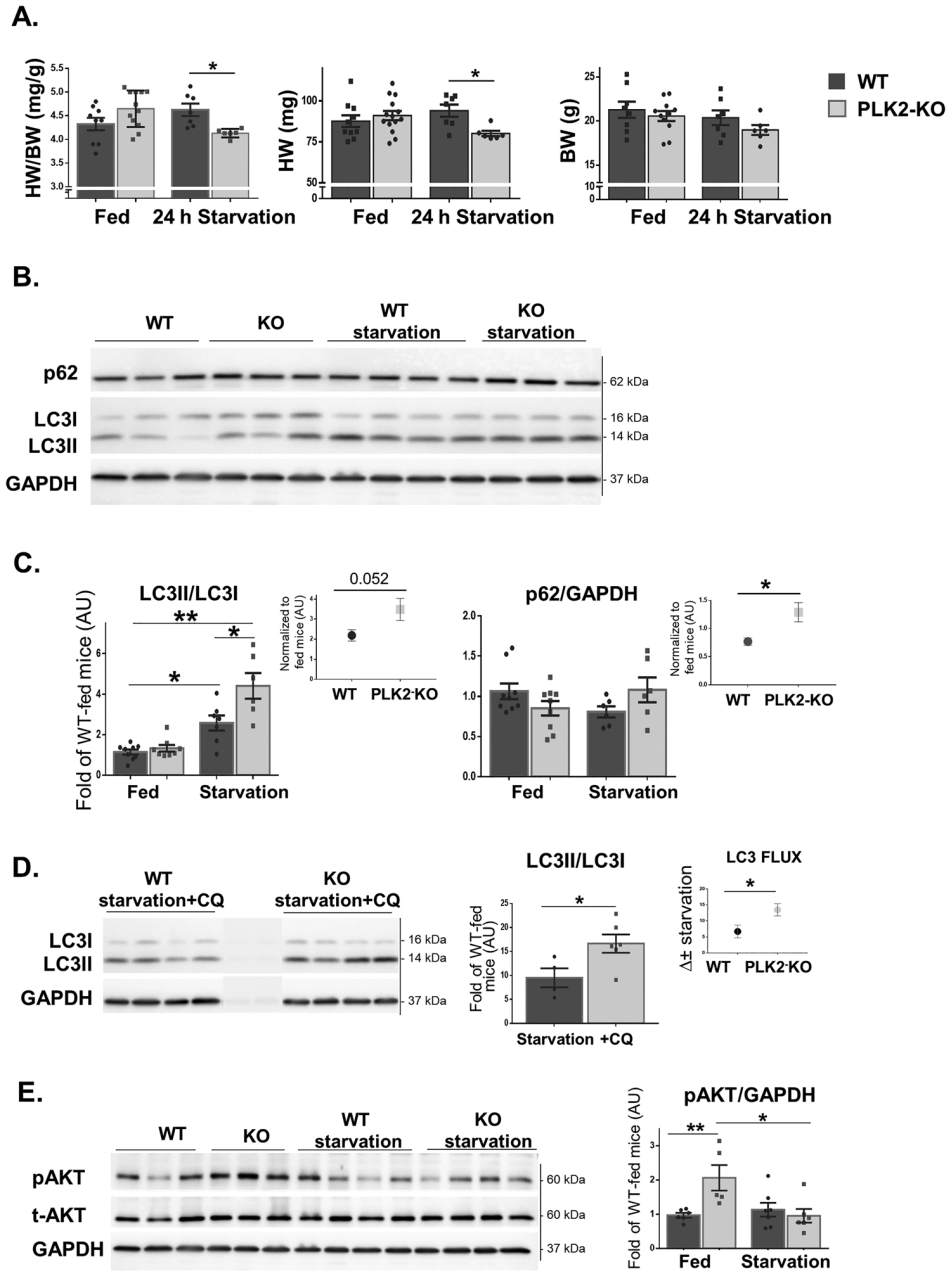
Excessive autophagy signaling following 24 h of starvation in PLK2-KO female mice. (**A**) Gravimetric analysis of heart weight (HW), body weight (BW), and the HW/BW ratio (n = 6–12 mice in each group) in female mice subjected to 24 h of starvation. (**B**). Representative example of WB results after 24 h of starvation for LC3I, LC3II, p62, and GAPDH with quantification (**C**). Autophagosome accumulation was calculated as the fold of starvation to untreated (fed) mice in each group (inset). (**D**) Mice were subjected to 24 h of starvation followed by 3 h of CQ (80 mg/kg). Representative example of WB results (left panel) with quantification (right panel). Autophagy flux was calculated as the delta between starvation + CQ and CQ in each group (∆ ± starvation, inset). (**E**) Representative example of WB results for phosphorylated AKT^473^ (pAKT), total AKT (t-AKT), and GAPDH and quantification of pAKT^473^ relative to GAPDH (n = 5–7 mice in each group). Statistical analyses were conducted with GraphPad Prism 6 as described in the Methods. *p < 0.05 and **p < 0.01.

During starvation, autophagy plays an essential role in maintaining cellular homeostasis in the heart^3^. Thus, we further investigated the effect of Plekhm2 deficiency on autophagy in female mice subjected to 24 h of starvation. CQ was intraperitoneally injected 3 h before extraction of the hearts. Starved PLK2-KO mice showed a decreased HW/BW ratio (*p* = 0.02; Fig. 3A), mainly due to a considerable reduction in heart weight. Both WT and PLK2-KO mice had an increase in the LC3II/LC3I ratio following starvation (*p* = 0.037 and *p* = 0.004, respectively; Fig. 3B, C). This increase was higher in starved PLK2-KO mice than in starved WT mice (*p* = 0.035). Following starvation, the p62/GAPDH protein level was not altered in PLK2-KO mice. However, when the p62 level of the starved mice was normalized to the fed state, PLK2-KO mice had a higher ratio compared with WT (*p* = 0.026, inset). An increase in autophagy markers represents stimulation of the autophagy process^3^. Thus, our results suggest that starved mice in both groups attempt to respond to starvation by increasing autophagy but that this adaptive response is dysregulated in PLK2-KO mice. Indeed, calculation of the autophagy flux revealed the additional accumulation of LC3II in PLK2-KO mice (Fig. 3D).

As already described above (Fig. 2C), there was a basal increase in pAKT^473^ in PLK2-KO mice compared with WT mice (*p* = 0.008; Fig. 3E). Starvation did not affect the pAKT^473^ level in WT mice but markedly decreased its level in PLK2-KO mice (*p* = 0.032), leading to pAKT^473^ starvation levels similar to those of WT.

### Cardiac fibroblasts are more vulnerable to Plekhm2 deficiency than cardiomyocytes

To further delineate the role of Plekhm2 in cardiac cells, we examined the effect of Plekhm2 ablation in vitro. Cardiac cells from neonates that were homozygous for the Plekhm2 floxed allele were transfected with adenoviruses expressing either CRE-recombinase (Ad-Cre) or GFP (Ad-GFP) for 5 days. *Plekhm2* mRNA (*plk2*) was markedly reduced to ~ 10% and ~ 20% in neonatal mouse cardiomyocytes (NMCMs) and neonatal mouse cardiofibroblasts (NMCFs), respectively (Supplementary Fig. S2 online). In agreement with the result in PLK2-KO mice, neither *plk1* nor *plk3* were overexpressed in Plekhm2-deficient cells (Supplementary Fig. S2 online).

**Figure 4:**
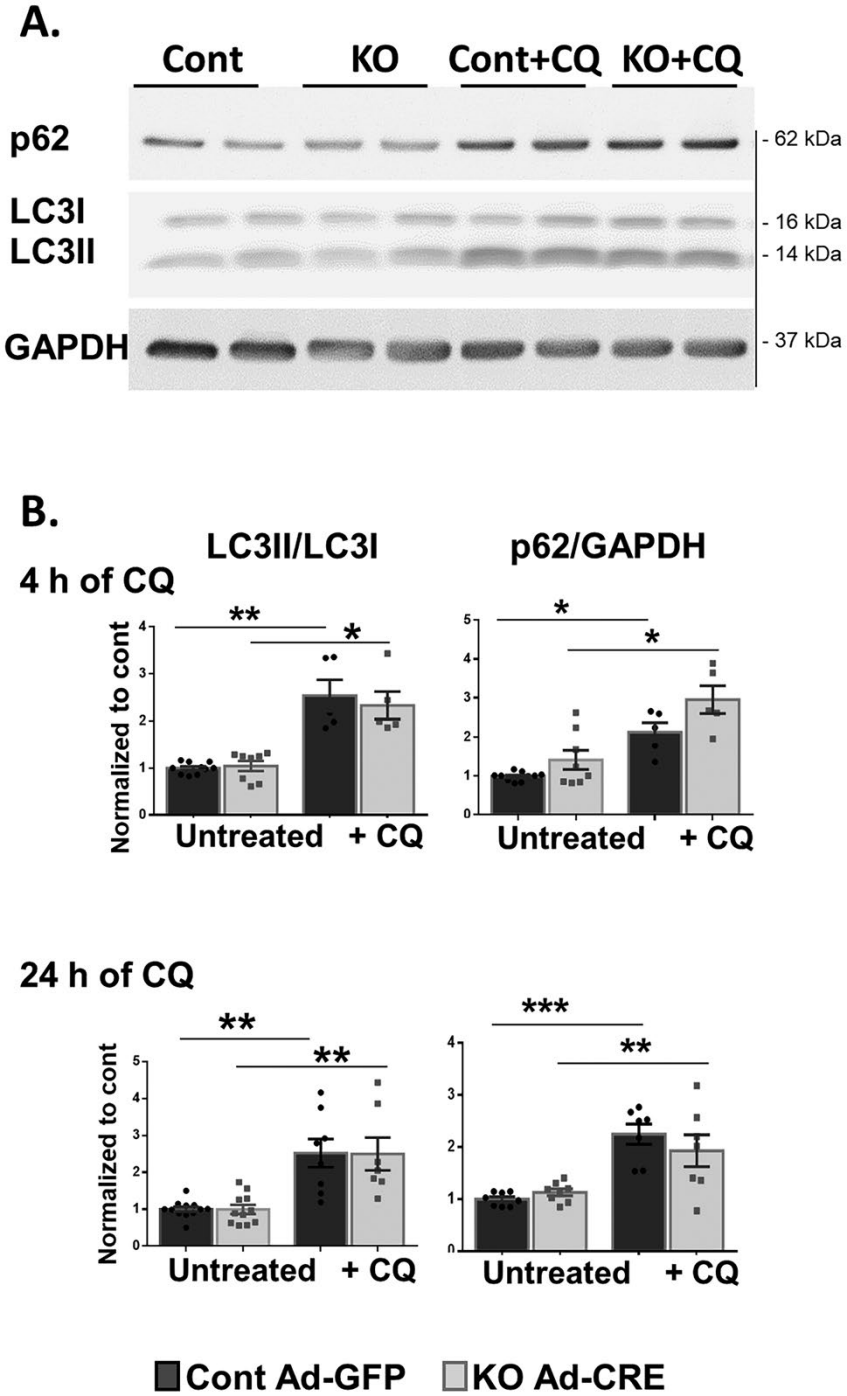
Accumulation of autophagosomes in neonatal cardiomyocytes following CQ treatment. NMCMs were transfected with Ad-Cre or Ad-GFP as control for 5 days and incubated with CQ (10 µM) 4 and 24 h before being harvested. (**A**) Autophagy was evaluated by WB assay for LC3I, LC3II, p62, and GAPDH. Quantification revealed the LC3II/LC3I ratio and p62/GAPDH elevation following 4 and 24 h of CQ treatment in KO and normal NMCMs (**B**). Statistical analyses were conducted with GraphPad Prism 6 as described in the Methods. *p < 0.05, **p < 0.01, and ***p < 0.001.

**Figure 5:**
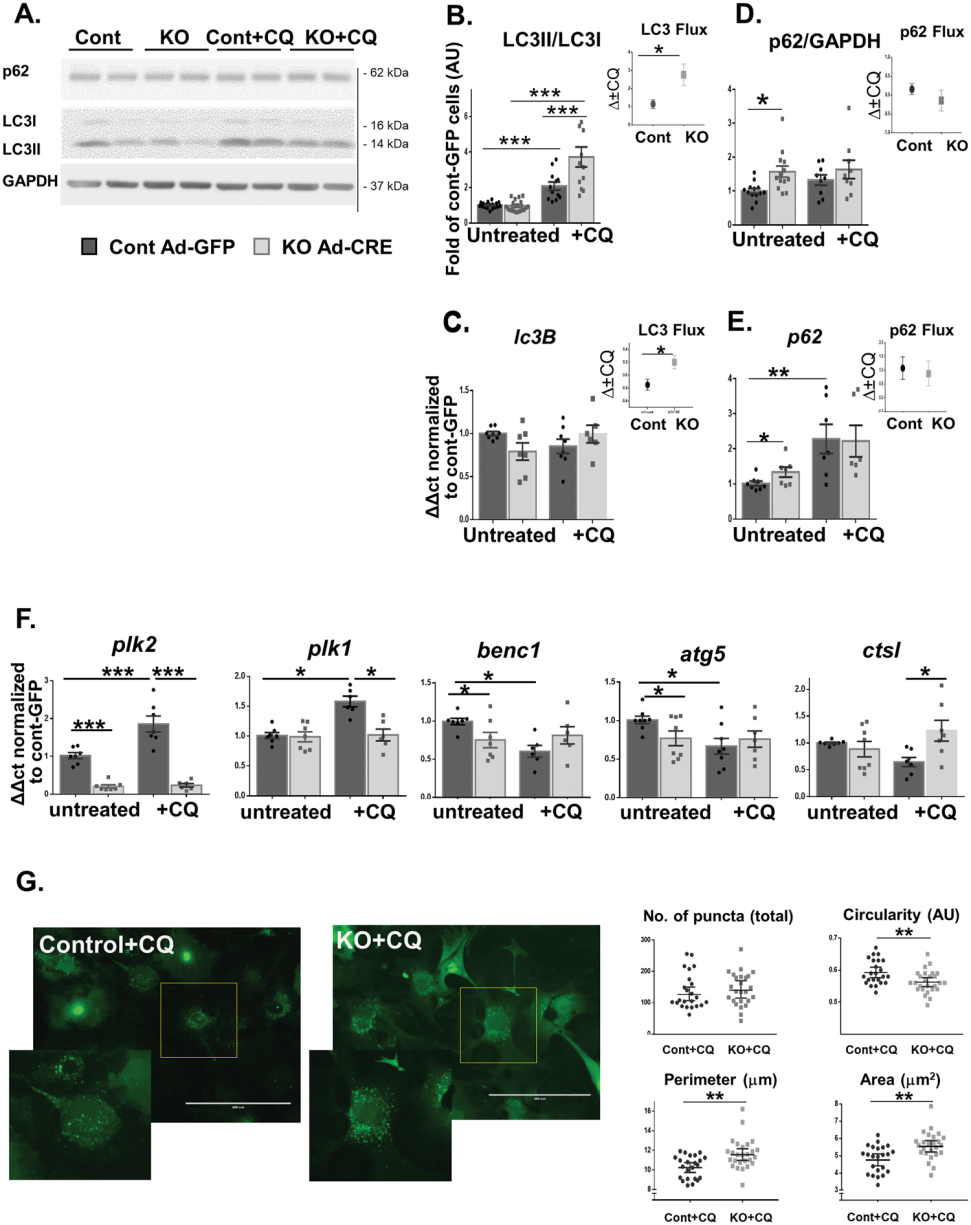
Accumulation of autophagosomes following CQ treatment in neonatal cardiac fibroblasts and mRNA expression of Plekhm and autophagy-related genes. NMCFs were transfected with Ad-Cre or Ad-GFP as control for 5 days and incubated with CQ (10 µM) 4 h before being harvested. (**A**) Autophagy was evaluated by WB assay for LC3I, LC3II, p62, and GAPDH. Quantification demonstrated elevations in the LC3II/LC3I ratio and p62/GAPDH following 4 h of CQ treatment in KO and normal NMCFs (**B**, **D**). Autophagy flux was calculated as the delta between CQ-treated and untreated cells in each group (insert); data were normalized to control cells (Ad-GFP) and are displayed as mean ± SEM (n = 5–12 wells in each group from 3–4 different isolations). Expression levels of various mRNAs in KO and control NMCFs subjected to 4 h of CQ treatment were examined with qPCR. (**C**). LC3II (*lc3B*). (**E**). p62 (*p62*). (**F**) Plekhm2 (*plk2*), Plekhm1 (*plk1*), and the autophagy-related genes Beclin1 (*becn1*), autophagy-related protein5 (*atg5*), and cathepsin L (*ctsl*). The mRNA expression levels in (**C**–**F**) were normalized to *gpdh* and are displayed as the fold of control (Ad-GFP) mRNA levels (n = 6–12 wells in each group from 3–4 different isolations). (**G**) NMCFs were infected with Ad-Cre (KO cells) or Ad-βGal as control (to avoid the green fluorescence of the GFP) for 5 days. Cells were transfected with Ad-LC3-GFP for 24 h followed by incubation with CQ (10 µM) for 4 h before being harvested. LC3 punctate fluorescence was examined with EVOS Floid® microscopy and accumulation and morphology parameters were quantified using ImageJ software in a total of 23 (cont-βGal) and 26 (KO-Cre) cells (8 and 7 wells, respectively, from two different isolations). Statistical analyses were conducted with GraphPad Prism 6 as described in the Methods. *p < 0.05, **p < 0.01, and ***p < 0.001.

As expected, autophagy flux was markedly increased following CQ treatment in both NMCMs (Fig. 4) and NMCFs (Fig. 5). However, while KO and control NMCMs demonstrated similar increases in the LC3II/LC3I ratio and p62 protein levels following 4 and 24 h of CQ treatment (Fig. 4), the KO-NMCFs exhibited an additional increase in the LC3II/LC3I ratio following 4 h of CQ treatment compared to the control treated cells (*p* = 0.0002; Fig. 5A, B). This finding was also emphasized in the autophagy flux calculation for the LC3 protein ratio (*p* = 0.02; Fig. 5B, inset). This increase could be at least partly attributed to the rise in the level of the *lc3B* mRNA flux (*p* = 0.02; Fig. 5C, inset) and the higher expression of LC3II protein normalized to GAPDH (*p* < 0.001, data not shown). At the basal level, we also detected an increase in the p62 protein and mRNA levels (Fig. 5D, E) in KO-NMCFs compared with the control, suggesting less degradation of autophagosomes and their greater accumulation in KO-NMCFs. However, no difference in autophagy flux was demonstrated for p62.

In the NMCFs, we further examined the mRNA levels of *plk2* and *plk1* and several additional genes related to autophagy. Both *plk2* and *plk1* were increased in the control cells following 4 h of CQ (*p* = 0.0001 and *p* = 0.013, respectively), suggesting their compensatory role in maintaining the normal autophagy process. This response was completely blunted in the KO-NMCFs (Fig. 5F). Furthermore, at baseline, the mRNA levels of *atg5* and *becn1*, which participate in the initiation phase of the autophagosome^24^, were lower in KO cells than in control (Fig. 5F); on the other hand, with CQ, their mRNA levels were decreased only in the control cells, with no change in the KO-NMCFs. Following CQ, a minor decrease was detected in control cells in *ctsl*, encoding cathepsin L, a lysosomal protease involved in protein turnover via activation of the autophagy-lysosomal pathway^25^; in contrast, in KO cells, its level was increased (*p* = 0.03). Calculation of the delta expression before and after CQ addition revealed reduced values for *atg5*, *becn1*, and *ctsl* in the control but not in the KO cells (*p* = 0.04, 0.002, and 0.006, respectively; Supplementary Fig. S3 online). These findings of abnormal autophagy flux in KO-NMCF cells indicate that Plekhm2 is indeed a vital component in their regulation of the normal autophagy flux.

To further assess the involvement of Plekhm2 in the autophagy flux, we examined LC3 puncta accumulation by transfecting the NMCFs with an adenovirus carrying LC3-GFP (Fig. 5G). Quantitative analysis indicated that the number of puncta did not differ between KO cells and controls but that the puncta were larger in KO cells, as indicated by perimeter and area measurements, and the circularity of the puncta in KO cells was lower.

### Plekhm2 deficiency during starvation differentially affects cardiac fibroblasts and cardiomyocytes

**Figure 6:**
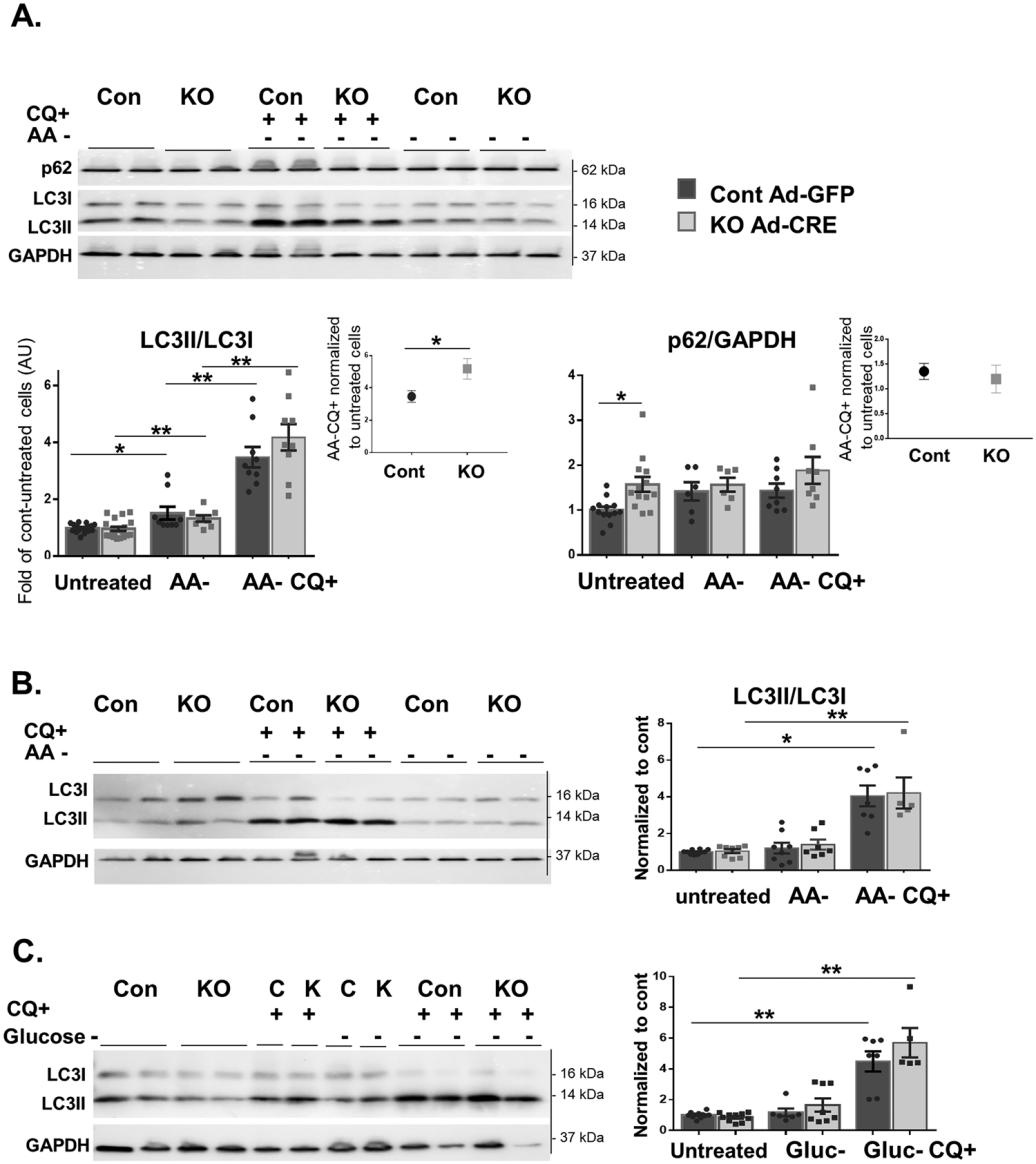
Starvation differentially activates autophagy in cardiac cells. (**A**) NMCFs (n = 7–14 wells) were transfected with Ad-Cre or Ad-GFP for 5 days. The cells were incubated overnight with serum-free medium followed by 4 h of incubation with EBSS (amino acid-free, AA^−^) medium with or without CQ (10 µM, CQ^+^). WB was performed for LC3II, p62, and GAPDH (upper panel) with quantification (lower panel). The increase in the LC3II/LC3I flux was calculated as the delta after both treatments (AA^−^ CQ^+^) to untreated cells (*p* = 0.033). B and C. NMCMs (n = 4–8 wells) were transfected with Ad-Cre or Ad-GFP for 5 days. Cells were incubated overnight with serum-free medium and subjected to EBSS (amino acid-free, AA^−^) medium (**B**) for 4 h or RPMI (glucose-free, Gluc^−^) medium (**C**) for 24 h with or without CQ (10 µM, CQ^+^) before being harvested. All results were obtained from 2–5 different cell isolations and were normalized to control (Ad-GFP). The results are displayed as mean ± SEM. *p < 0.05, **p < 0.01, and ***p < 0.001 by Kruskal–Wallis test with Dunn’s multiple comparison post-test or Mann–Whitney nonparametric test as described in the Methods.

To explore the effect of starvation, we induced autophagy in NMCFs via amino acid deprivation and exposed the cells to CQ for 4 h to measure autophagy flux. Following amino acid deprivation, there was a similar minor increase in the LC3II/LC3I ratio in both KO and control cells (Fig. 6A). However, administration of CQ yielded a higher increase in the LC3II/LC3I ratio in the KO cells than in the controls (*p* = 0.033; Fig. 6A, inset). Higher expression of p62 protein was also noted in untreated KO cells, as also described earlier (Fig. 5D), but with no alterations following exposure to amino acid deprivation and CQ. Interestingly, no difference was found between KO and control NMCMs exposed to 4 h of amino acid deprivation (Fig. 6B) or 24 h of glucose deprivation (Fig. 6C), with or without CQ.

### Plekhm2 KO hearts are less susceptible to AngII

**Figure 7:**
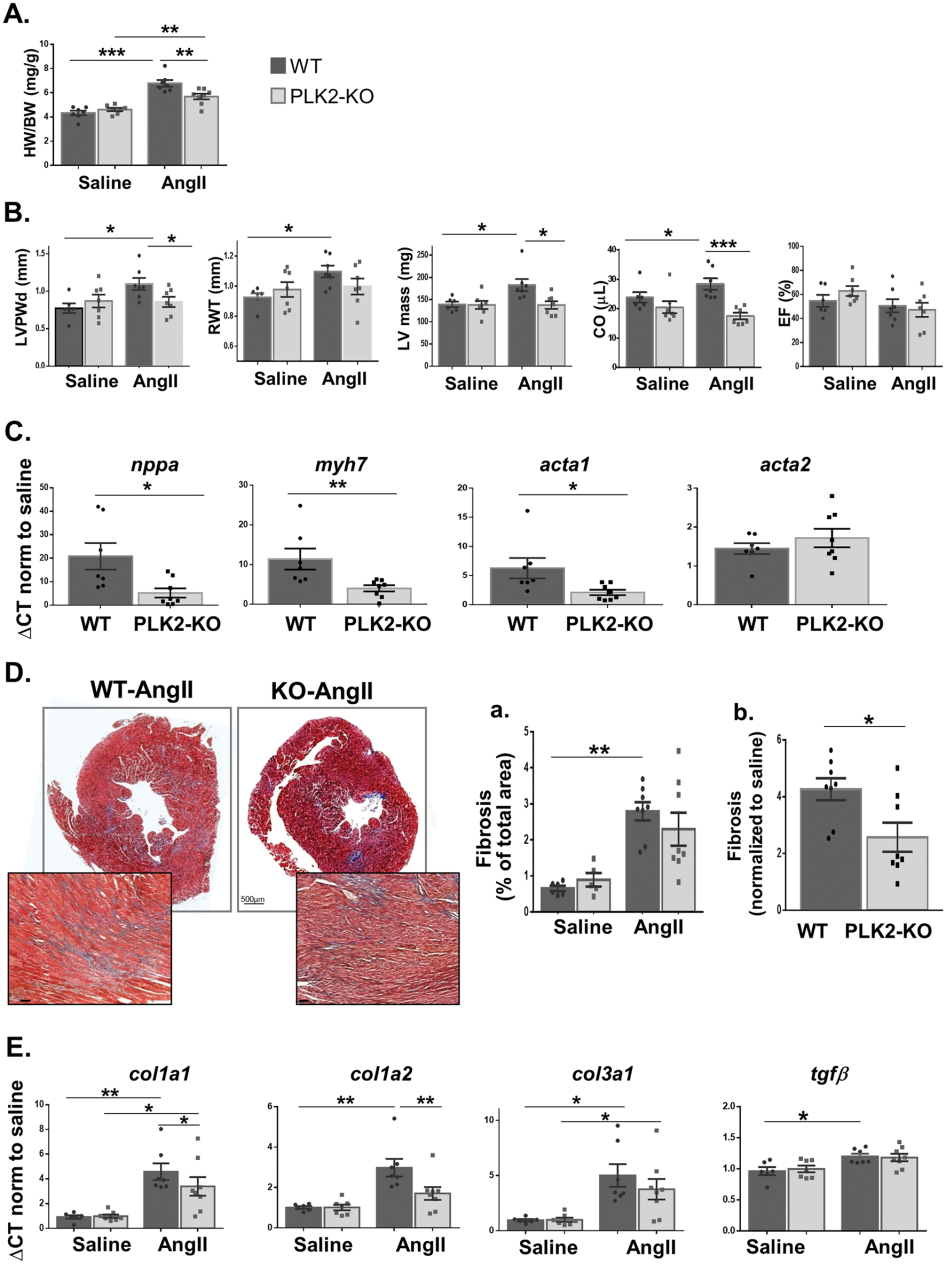
Plekhm2 deficiency attenuates AngII-induced cardiac hypertrophy in mice. Mice (males) were implanted with Alzet osmotic pumps containing saline (n = 6–7) or Angiotensin-II (AngII; 2 mg/kg/day, n = 7–8) for 2 weeks. Gravimetric analysis of the heart weight/body weight (mg/g) ratio (**A**) and echocardiographic parameters (**B**) were both assessed 2 weeks after implantation and revealed cardiac hypertrophy in WT mice in response to AngII. (**C**) mRNA levels of genes related to cardiac stress: natriuretic peptides A (*nppa*), myosin heavy chain7 (*myh7*), actin, alpha skeletal muscle (*acta1*), and actin, aortic smooth muscle (*acta2*). (**D**) Paraffin-embedded sections of whole heart were stained with Masson’s trichrome. Cardiomyocytes are stained in red while the fibrotic area is stained in blue. Fibrosis was calculated as the percentage of the fibrotic area from the total area of the heart section and the results are normalized to WT saline (**a**). The difference between WT and PLK2-KO mice treated with AngII is emphasized when the results are normalized to saline treatment in each group (**b**). (**E**). mRNA levels of genes related to fibrosis: collagen alpha-1(I) chain (*col1a1*), collagen alpha-2(I) chain (*col1a2*), collagen alpha-1(XIII) chain (*col3a1*), and transforming growth factor beta (*tgfβ*). The results for mRNA levels are displayed as the fold of saline-treated mice (∆CT) due to high variability in the baseline results. Statistical analyses were conducted with GraphPad Prism 6 as described in the Methods. *p < 0.05, **p < 0.01, and ***p < 0.001.

AngII is a potent neurohormonal modulator involved in cardiac remodeling^26^. Thus, to further assess the possible role of Plekhm2 in maintaining cardiac function, we evaluated the effect of long-term exposure to AngII on PLK2-KO mice. We hypothesized that the cardiac phenotype of global PLK2-KO mice might be further impaired under chronic neurohormonal stress conditions. Following exposure to AngII for 2 weeks, gravimetric analysis demonstrated a lower BW, which was not significantly different between KO and WT mice (Supplementary Table S4 online). In WT mice, AngII-induced cardiac hypertrophy was characterized by an increased HW (*p* < 0.001) and HW/BW ratio (p < 0.001) (Supplementary Table S4 online). Surprisingly, PLK2-KO mice were less sensitive to AngII, as indicated by the smaller HW (WT vs. KO, 167.7 ± 8.6 mg vs. 128.3 ± 3.8 mg; *p* < 0.001) and reduced HW/BW ratio compared with treated WT mice (Fig. 7A and Supplementary Table S4 online).

Reduced sensitivity of the PLK2-KO heart to AngII was also confirmed via echocardiographic measurements (Supplementary Table S4 online). In the WT mice, increases in LVPWd (*p* = 0.027), RWT (*p* = 0.049), and LV mass (*p* = 0.038) were noted following exposure to AngII, with no change in the EF (*p* = 0.9). On the other hand, PLK2-KO mice showed reduced hypertrophy following AngII, as indicated by the lower LVPWd (*p* = 0.039) and LV mass (*p* = 0.007) compared with AngII-treated WT mice (Fig. 7B and Supplementary Table S4 online). The cardiac function (i.e., fractional shortening and EF) of the PLK2-KO mice in response to AngII was unaltered compared with WT mice. However, stroke volume and cardiac output were smaller, again reflecting the smaller cardiac size in AngII-treated PLK2-KO mice versus WT. AngII induced an increase in the cardiac stress-related genes *nppa*, *myh7*, and *acta1* in WT treated mice, but this maladaptive response was reduced in PLK2-KO mice (Fig. 7C).

Finally, we characterized the role of Plekhm2 in the fibrotic response to AngII via ventricular Masson’s trichrome staining. Consistent with the above findings indicating an attenuated hypertrophic response in PLK2-KO mice, we noted a reduced fibrotic response in AngII-treated PLK2-KO mice relative to AngII-treated WT mice (*p* = 0.0018; Fig. 7Da). This attenuated fibrotic response of the PLK2-KO mice was further confirmed when the AngII results were normalized to the saline results (*p* = 0.020; Fig. 7Db). Moreover, while genes related to fibrosis, including *col1a1, col1a2*, *col3a1*, and *tgfβ*, were all induced by AngII in the WT mice, attenuated induction of *col1a1* (*p* = 0.05) and *col1a2* (*p* = 0.009) was evident in AngII-treated PLK2-KO mice (Fig. 7E).

Overall, the above results indicate that, in contrast to our original assumption, ablation of Plekhm2 in the mouse results in reduced sensitivity to pathological hypertrophy induced by neurohormonal stress.

## Discussion

**Figure 8:**
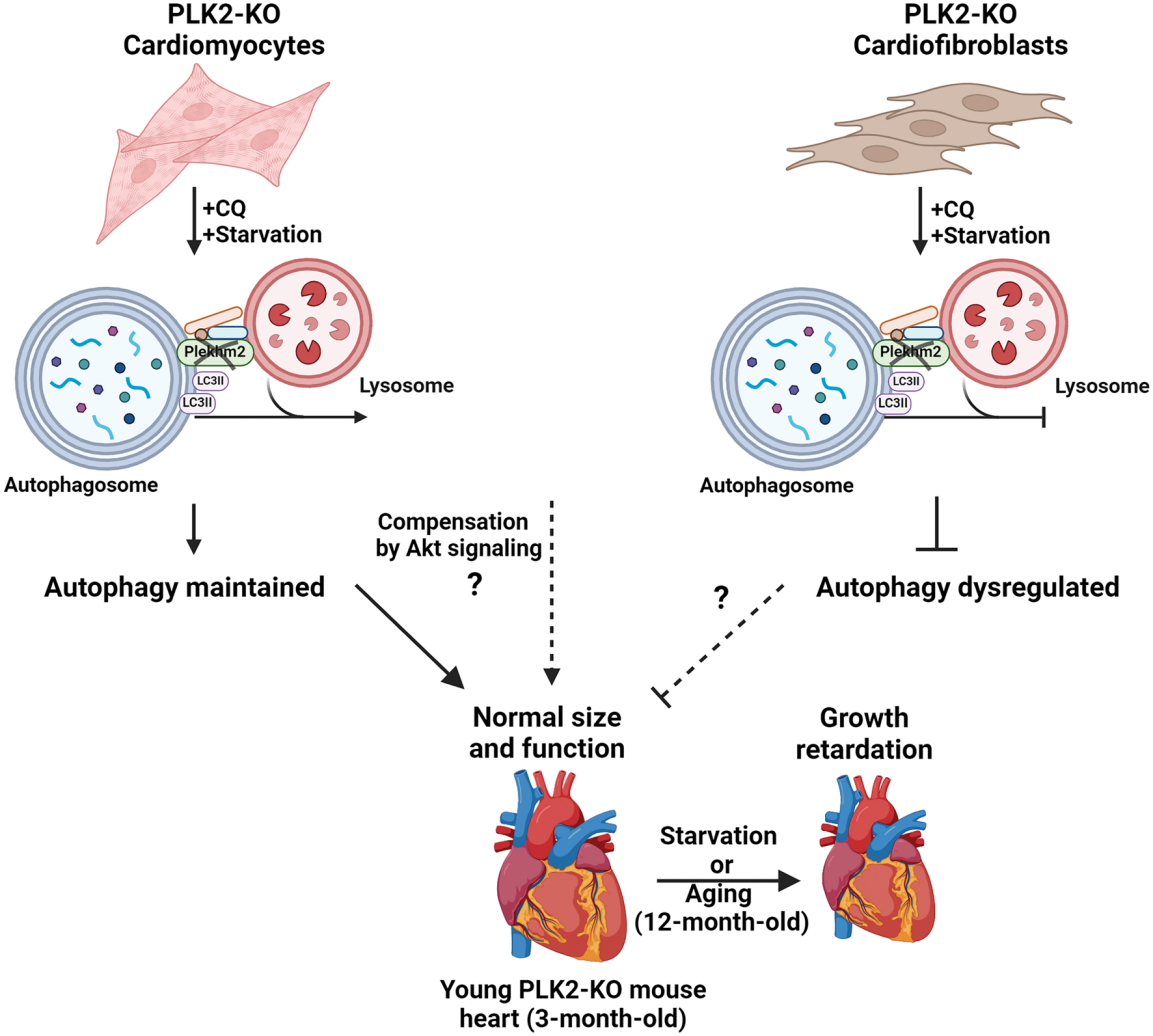
Postulated role of Plekhm2 in the murine vs. the human heart. Young PLK2-KO mice exhibit no difference in cardiac function or autophagy markers but demonstrate higher basal Akt phosphorylation. Growth retardation was observed following starvation and in old PLK2-KO mice (12-month-old). Impaired autophagy was also noted in vitro in PLK2-KO cardiofibroblasts but not in cardiomyocytes. Overall, the absence of Plekhm2 in mice appears to promote compensatory mechanism(s) enabling the heart to manage functionally even in the presence of neurohormonal stress without detrimental consequences. Importantly, while PLEKHM2 loss of function mutations are clearly associated with a DCM phenotype in human^9,16^ and autophagy derangement in patient's primary fibroblast^9^ as well as in iPSC-induced cardiomyocytes^17,46^, autophagy derangement could not be identified in murine cardiomyocytes. Whether this overt difference explains the absence of DCM phenotype in the PLK2-KO mice remains to be determined. Figure was created with BioRender.com.

In this study we examine the roles of Plekhm2 in the murine heart using PLK2-KO mice and cultured primary cardiac cells with ablated Plekhm2 expression. The main motivation for our study steams from the fact that *PLEKHM2* loss-of-function mutations were associated with early onset DCM in two independent studies^9,16^, as well as abnormal lysosomal localization and impaired autophagy in the fibroblasts of an inflicted indivisual^9^. Our main findings indicate that murine Plekhm2 is essential for normal cardiac autophagy following nutrient deprivation and aging. Plekhm2-deficiency in cultured cardiofibroblast impaired autophagy, but the autophagy in murine cardiomyocytes was not critically dependent on Plekhm2. Surprisingly, we found that ablation of Plekhm2 did not lead to cardiac dysfunction and even reduced the murine heart sensitivity to pathological hypertrophy following neurohormonal stress, a finding that seems to be in clear contrast with the human cardiac phenotype described above. These complex findings are discussed in details below and summarized in Fig. 8.

Plekhm2 is involved in lysosomal transport via coupling to the microtubule motor kinesin-1 via the small GTPase Arl8 and the BORC complex, which enables autophagosome-lysosome fusion^14,15,27^ and lysosomal trafficking^28^. We hypothesized that Plekhm2 deficiency would impair autophagosome-lysosome fusion, thereby accelerating the accumulation of autophagosomes in the heart and leading to cardiac dysfunction. However, our results indicate that, at a young age, the absence of Plekhm2 does not affect cardiac function (Supplementary Tables S1, online) and that the autophagy process seems to remain balanced (Fig. 1), except for a slight increase in p62 flux vs. WT siblings. This minor increase may suggest a minor interruption to the process of autophagosome degradation in young PLK2-KO mice.

Plekhm1 and Plekhm3 were recently reported to participate in cardiac function under stress conditions in mice. Zhang et al.^18^ showed that CD38 regulates autophagic flux by regulating the core proteins Rab7 and Plekhm1. Loss of Rab7/Plekhm1 impaired the fusion of autophagosomes and lysosomes, resulting in the accumulation of autophagosomes in the myocardium and consequent cardiac dysfunction under hypoxic/ischemic conditions. Plekhm3 is reported to be modulated by miR-320 during heart failure after transverse aortic constriction and following AngII administration^19^. While Plekhm1 and Plekhm3 were found to be important in the heart, their mRNA levels were lower than that of *Plekhm2* and were not altered in PLK2-KO mice, indicating that they do not compensate for the absence of Plekhm2. Nevertheless, in the mouse heart, the function of Plekhm1 and/or Plekhm3 possibly overlaps that of Plekhm2, leading to the relatively mild phenotype that we observed compared with the loss of function of this gene in the human heart, a phenotype that is already severe in young children and in early adulthood. Further studies are needed to explore this possibility in detail based on the current findings.

Autophagy is a vital process for maintaining cardiovascular homeostasis during aging. Several studies have implicated reduced autophagy in the deterioration of cardiovascular function and increased susceptibility to cardiovascular disease upon aging^29^. In our study, we demonstrated growth retardation and a lower HW/BW ratio and HW to tibia length ratio in 12-month-old PLK2-KO mice (Supplementary Tables S1 online). We also noted changes in some physiological parameters with aging, which mainly reflected the smaller cardiac size (Supplementary Fig. S1 and Tables S1 and S3). The LC3II protein level was upregulated in aged PLK2-KO mice compared with aged WT mice (Fig. 2), suggesting dysregulation of autophagy in KO mice and a slight cardiac dysfunction with aging. In previous work, cardiac dysfunction was demonstrated in older mice (at least 18 months old) through LV systolic dysfunction, a reduced EF, hypertrophy, and fibrosis, as well as an increase in LC3II and pAKT protein levels^29–31^. We can speculate that the phenotype of the middle-aged (12-month-old) PLK2-KO mice that we describe here might lead to more overt cardiac dysfunction over time. However, because we did not test older mice, this possibility remains unanswered. Additional studies measuring the autophagic flux in 12-month-old mice as well as in older mice are needed to firmly establish how aging affects authophagy in Plekhm2-deficiency heart.

Starvation is a classic model to demonstrate the role of autophagy in the cellular homeostasis of the heart^3,32^. Although the ratio was higher in PLK2-KO mice, both WT and PLK2-KO mice showed an increase in the LC3II/LC3I ratio after 24 h of starvation, which was accompanied by a significant reduction in heart weight (Fig. 3). The increased levels of the autophagy proteins LC3II and p62 in PLK2-KO hearts suggested an accumulation of autophagosomes, possibly due to impaired degradation of the autophagosome rather than increased formation^5,33^. Excessive autophagy due to an imbalance between autophagosome formation and lysosomal degradation causes a massive accumulation of autophagosomes, which subsequently affects cellular function and contributes to cardiotoxicity through autophagic cell death^7,34,35^. Jia et al.^15^ showed that KO of BORC, which is part of the Plekhm2-Arl8-kinesin1 multicomplex, influences lysosomal positioning and increases the levels of the autophagy proteins LC3B-II and SQSTM1/p62. Therefore, BORC-KO impairs both the interaction between and fusion of autophagosomes with lysosomes, suggesting that the LC3B-II accumulation is largely the result of reduced lysosomal degradation rather than enhanced autophagy initiation. Lysosome-associated membrane protein 2 (LAMP2)-deficient mice also exhibit excessive accumulation of autophagic vacuoles and impaired autophagic degradation of long-lived proteins, resulting in cardiomyopathy^36,37^. Furthermore, Nah et al.^35^ showed that Rubicon, a negative regulator of autophagy, is upregulated during stress and blocks autophagosome-lysosome fusion, leading to the accumulation of autophagosomes and facilitating autophagy-dependent cell death. Impaired autophagy or massive activation are both detrimental to the heart and may cause cellular dysfunction and death^7^. Taken together, we suggest that the smaller hearts and growth retardation observed in stressed PLK2-KO mice may be partially explained by the excessive autophagosome accumulation that culminates in autophagic cell death.

The balanced autophagy in young PLK2-KO mice and its dysregulation following stress raise the possibility of a compensatory mechanism that prevents the pathological consequences of Plekhm2 deficiency at a young age. Several molecular pathways that facilitate cardiovascular health are associated with autophagy function. The mTOR complex and its associated pathways play a key regulatory role in cardiovascular physiology and pathology^38,39^. This process is activated by nutrients and growth factors and is inhibited during starvation, leading to autophagy activation and cell survival^3,6,40^. The mTOR/AKT pathway is associated with cardiomyocyte protection during ischemic injury and preserved cardiac structure and function. It participates in compensatory growth in response to mechanical stress^3,41^, as well as protection during hypoxia-induced injury^42^ and pressure overload-induced cardiac hypertrophy^43^. In our study, we identified an increase in phosphorylated AKT^473^ in young PLK2-KO mice (Fig. 2C and 3E). Because AKT is a negative regulator of autophagy, we speculated that activated AKT^473^ would inhibit excessive autophagy and maintain cardiac homeostasis in young PLK2-KO mice. Interestingly, aged WT mice showed elevated AKT activity, in line with previous studies that demonstrated a role for the PI3K/AKT pathway and autophagy in cardiac aging^30,31^. The short-term activation of AKT may promotes physiological changes and protection from myocardial injury; whereas, its long-term activation may cause pathological outcome and heart failure. In our study, PLK2-KO mice showed less activation of AKT with aging that may reflect an increase in the autophagy pathway in order to maintain cardiac homeostasis.

However, these results are causative and we need further studies to reveal the association between AKT signaling and PLEKHM2-deficiency in the heart. Such studies are beyond the scope of the present manuscript. It should be noted that regulation of autophagy is extremely complex process involving various signaling pathways. The mTOR activity is a core checkpoint of autophagy influenced by AKT and AMPK, both playing an essential role in autophagy and in the maintenance of cardiac homeostasis^44^. Ren, J et al.^31^ showed that Akt2 ablation protects against cardiac aging through restored Foxo1-related autophagy and mitochondrial integrity while AKT2-AMPK double ablation predisposes cardiac aging possibly related to compromised autophagy and mitophagy^44^. Thus, it appears that autophagy regulation may be physiological or pathological largely depending on the nature and extend of the cellular stress^45^.

We further characterized the role of Plekhm2 deficiency in NMCMs and NMCFs. Our results showed no difference in the autophagy flux between KO and control NMCMs under basal conditions (Fig. 4) and following amino acid (Fig. 6B) or glucose (Fig. 6C) deprivation. In contrast, KO-NMCFs demonstrate several changes in the autophagy process (Figs. 5 and 6A), indicating accumulation of autophagosomes and a defect in the autophagy process. Cathepsin L, an important member of the lysosomal protease family, plays a valuable role in protecting against cardiac dysfunction following pressure overload via activation of the autophagy-lysosomal pathway^25^. An increase in the mRNA level of cathepsin L (*ctsl*) in the CQ-treated KO-NMCFs suggests a compensatory effect of lysosomes that is triggered by the autophagy derangement. The lower mRNA levels of *atg5* and *becn1* (Fig. 5F), participants in the initiation phase of the autophagosome^24^, in KO cells with no changes following CQ support the autophagy dysregulation in NMCFs. Finally, the increase in the mRNA levels of *plk2* and *plk1* in response to CQ in control cells accentuates their possible involvement in normal NMCF autophagy (Fig. 5F). Based on our results it appears that Plekhm2 deficiency in the murine heart can affect NMCF but not NMCM autophagy. In future studies, a co-culture approach may be of value to delineate if interactions between NMCF and NMCM can regulate the autophagic flux in both cell types.

The above in vitro finding that points to differential effect of Plekhm2 loss of function in regard to autophagy dysregulation is surprising. It appears that the murine cardiomyocytes might have a compensatory mechanism making them resistance to Plekhm2 loss of function. Interestingly, although the global PLK2-KO mice demonstrated growth retardation and smaller hearts, their physiological findings are not consistent with the severe DCM found in humans with PLKHM2 loss of function^9,16^. Moreover, magnetic resonance imaging of one such patient revealed rapid and massive loss of myocardial tissue over a relatively short period^46^. Thus, it is possible that, while PLEKHM2 is indeed vital in human cardiomyocytes, it is compensated by other genes/cellular components in murine cardiomyocytes. The recent report that induced pluripotent stem cell-derived cardiomyocytes with PLEKHM2-KO show impaired autophagy in association with reduced contractility and impaired calcium handling^17^ are clearly supporting this possibility.

PLK2-KO mice did not demonstrate a DCM phenotype either spontaneously or in the presence of AngII, further supporting the notion that in contrast to humans, global loss-of-function of the *Plekhm2* in mice is not leading to DCM phenotype. At this point it is hard to directly reveal the molecular mechanism/s leading to this unexpected finding. It is important to note that dysregulation of autophagy may play an important role in AngII-induced cardiac stress although conflicting reports exists regarding this issue^26^. Interestingly, several studies (e.g.,^47^) demonstrate a cooperation between the ubiquitin–proteasome system (UPS) and lysosomal-autophagy pathway. In this regard, our results may also raise the possibility that the UPS, rather than the autophagy-lysosome pathway, is responsible for maintaining cardiac function following AngII when the lysosomal-autophagy process is dysregulated. However, further studies are required to test these possibilities in detail.

In conclusion, our findings are compatible with a role for Plekhm2 in cardiac autophagy. However, it appears that the autophagy and the viability of murine cardiomyocytes are not critically dependent on the expression of Plekhm2, possibly because of the activation of AKT signaling and/or other compensatory pathways. This may explain the discrepancy between the malignant DCM phenotype of human PLEKHM2 loss of function and that of our murine KO model.

## Materials and methods

### Global Plekhm2 KO model

The study was conducted in strict accordance with the Guide for the Care and Use of Laboratory Animals of the National Institutes of Health and in accordance with ARRIVE guidelines . All experiments were approved by the institutional ethics committee of Ben-Gurion University of the Negev, Israel (Protocols IL4108-2016 and IL5908-2020D).

Plekhm2tm1a/(EUCOMM)Wtsi mice with a “knockout first allele” (a kind gift from Dr. Stephane Meresse^48^) contain a cassette between exons 7 and 8 of Plekhm2 with a splice acceptor site, Lacz and neo genes, and a PolyA signal at the 3'-end of the cassette. The cassette is flanked by FRT sequences and a loxP site. Another loxP site is inserted between exons 8 and 9 (Supplementary Fig. S4 online). During transcription, the cassette with the Lacz and neo genes is spliced to exon 7 of Plekhm2, resulting in cessation of transcription because of the PolyA sequence. Therefore, Plekhm2 protein is not expressed in these mice. The generation of Plekhm2 floxed mice for Plekhm2 KO cell cultures is described in detail in the Supplementary Materials and Methods.

### Genotyping

PCRs were conducted with specific primers (designed by the Emma Consortium)—forward Plekhm2_44411, TCCTCACTGG AAAGCAGCAC; reverse Plekhm2_44411, CAGGCAGGGT GAGTTTGATG; and CAS_R1_Term, TCGTGGTATC GTTATGCGCC—according to the schematic (Supplementary Fig. S4 online) to detect each genetic manipulation. Figures S4 C and D provide examples of the genotyping results of the respective mice and cell cultures. The DNA marker was GeneRuler 1 kb Plus DNA Ladder (Thermo Fisher Scientific).

### In vivo models

While the basic sections of the study were performed in both males and females, other experiments were limited to one sex only. Thus, in all of the Results sections, the sex of the mouse in each experiment is explicitly mentioned.

#### Starvation

Three-month-old female mice were starved via food deprivation for 24 h with free access to water. For analyses of the autophagic flux in vivo, we applied intraperitoneal CQ 80 mg/kg or vehicle (NaCl 0.9%) 3 h before heart extraction.

#### AngII osmotic mini-pumps

Three-month-old male mice were implanted with osmotic mini-pumps (ALZET pump Model 2004; Alzet, CA, USA). The mini-pumps constantly infused AngII (dissolved in saline) at a rate of 2 mg/kg/day or vehicle (saline) for 14 days. Under deep pentobarbital anesthesia, mouse ventricles were quickly extracted and immersed in cold PBS (Mg^2+^ and Ca^2+^ free), weighed, divided into three sections, and frozen immediately in liquid nitrogen or prepared for histological sectioning.

### Echocardiography

We performed echocardiographic measurements under light isoflurane anesthesia and strict temperature control using a Vevo 3100 ultrasound (FUJIFILM VisualSonics, Toronto, Canada) as previously described by us^49,50^. All measurements were performed in a blinded manner by a skilled technician.

### Histological analysis

Transverse sections from the mid-base of hearts were fixed in 4% paraformaldehyde for 24 h, embedded in paraffin, and cut into 5-µm-thick slices. Masson’s trichrome staining was conducted according to the manufacturer’s instructions (Bio Optica, Milan, Italy). The stained sections were inspected with a digital slide scanner (Pannoramic MIDI II; 3DHISTH, Budapest, Hungary) and analyzed by ImageJ software using the Color Deconvolution Masson’s trichrome plugin, which specifically marks blue (fibrotic) areas. Large vessels and false-positive staining were excluded. Fibrosis was calculated as the percentage of fibrotic area from the total area of the heart section (stained in red). All of the histological analyses were conducted in a blinded manner.

### Plekhm2 ablation in primary cultures of neonatal mouse heart cells

Primary cultures of NMCMs and non-cardiomyocytes (mainly cardiac fibroblasts (NMCFs)) were isolated via the enzymatic dissociation of hearts from 0 to 3-day-old neonate Plekhm2^floxed/floxed^ mice. Mice were deeply anasthesized with isoflurane and the hearts were collected into ice-cold M-199 medium, gently washed, and cut into 1–2-mm^3^ pieces in calcium- and magnesium-free PBS (both from Biological Industries, Inc., Haifa, Israel). The cardiac tissue pieces were subjected to 5–7 cycles of enzymatic digestion using 0.12 mg/mL pancreatin (Sigma (Merk), MO, USA) and 45 units/mL of collagenase type 2 (Worthington, Lakewood, NJ, USA) dissolved in ADS buffer (in mM: 116 NaCl, 1 NaH_2_PO_4_, 5.5 glucose, 5.39 KCl, 1 MgSO_4_ × 7H_2_O, 20 HEPES, pH 7.4). Fractions from all cycles were combined, centrifuged for 5 min at 4°C and 600 × *g*, and resuspended in M-199 supplemented with 5% fetal bovine serum (FBS; Biological Industries Inc.). The cell suspension was pre-plated for 75 min on an uncoated plate to separate the non-cardiomyocytes. Next, the non-attached cells (mostly NMCMs) were collected and plated in 12-well gelatin-coated plates for 24 h before the addition of new plating medium. The attached cells (mostly NMCFs) were maintained in DMEM/15% FBS for growth and a single passage. The cells were scraped and plated on gelatin-free 6-well plates in DMEM/10% FBS medium for further growth and transduction^49,51^.

### Adenovirus transduction

After 48 h in culture, isolated cells were transduced with 75 MOI (NMCMs) or 110 MOI (NMCFs) of purified (Vivapure, Gottingen, Germany) adenovirus expressing either GFP (Control) or CRE-recombinase (a kind gift from Prof. Itzhak Kehat) for 2 h in serum-free medium to induce Plekhm2 KO. In the experiments in which adenovirus carrying GFP-LC3 gene was applied (see below), adenovirus carrying β-galactosidase served as control. After 2 h, medium supplemented with 5% FBS (for NMCMs) or 10% FBS (for NMCFs) was added for an additional 24 to 48 h. DNA samples for PCR were obtained after 5 days to verify the deletion of *Plekhm2* gene. Expression of fluorescent GFP was also used to verify the transfection quality.

### Autophagy induction in culture

Autophagy was stimulated via deprivation of glucose for 24 h (RPMI 1640) or amino acids for 4 h (Earle’s Balanced Salt Solution [EBSS]) in serum-free medium. Control cells were incubated with serum-free medium for 24 h (M-199 for NMCMs and DMEM for NMCFs). CQ (10 µM) was added to the medium 24 or 4 h before the end of the experiment to determine the autophagy flux^20,21^. Autophagy flux was also examined by transfecting the cells with adenovirus carrying GFP-LC3 gene^52^ (a kind gift from Prof. Lorrie Kirshenbaum). NMCFs were infected with Ad-Cre (110 MOI) or Ad-β-gal (120 MOI) for 3 days, followed by transfection with Ad-GFP-LC3 (75 MOI) for another 48 h. Four hours before the end of the transfection, the cells were incubated with serum-free medium with or without CQ.

### Western blotting

Cells or heart tissues were homogenized in RIPA buffer containing protease and phosphatase inhibitors (ROCH®) and prepared for WB as previously described^49,51^. The samples (20 µg each) were separated on a 12.5% polyacrylamide gel and transferred to polyvinylidene difluoride (PVDF) membrane (Bio-Rad). Membranes were incubated overnight at 4°C with the following primary antibodies: LC3B (#2775), pAkt^ser473^ (#4060), and t-AKT (#9272), all from Cell Signaling Technology, and p62 (#ab56416, Abcam). GAPDH (#MAB374, Millipore) served as loading control. Membranes were washed three times for 5 min each and incubated with tissue-specific horseradish peroxidase-conjugated secondary antibodies: anti-mouse (#7076) and anti-rabbit (#7074), both from Cell Signaling Technology. The signal was detected with chemiluminescence substrate (WESRAR ɳC XLS100, 0500, or WESTAR SUPERNOVA XLS3, 0100; Bio-lab) using a FUSION SOLO X (Vilber Lourmat) apparatus and quantified with ImageJ software. Each heart sample was subjected to 2–4 different WB runs. The results were normalized to the control and the average of each sample was used for statistical analysis.

### Gene expression analysis by real-time fluorescent qPCR

Total RNA was extracted from cardiac cells or hearts and real-time qPCR was performed as previously described^49^. Relative gene expression was calculated by the efficiency 2^−ΔΔCT^ method or as relative measurements (2^−ΔCT^) with the expression of the genes of interest (Supplementary Table S5 online) normalized to that of the housekeeping gene GAPDH. Each sample was tested in triplicate.

### Statistical analysis

Values are expressed as mean ± standard error of the mean (SEM). Statistical analysis was performed using Prism 6.0 (GraphPad Software, San Diego, CA, USA). Comparisons between control and PLK2-KO groups were performed using an unpaired Student's t-test. When the n number was lower than 6 or the Shapiro–Wilk normality test was < 0.05, the Mann–Whitney test was performed instead. Comparisons between treatments were performed by one-way ANOVA with multiple comparisons. When the n number was lower than 6 or the Shapiro–Wilk normality test was < 0.05, the Kruskal–Wallis test with Dunn’s multiple comparison post-test was performed instead. The specific tests used are mentioned in the legend of each figure. The criterion for significance was set at *p* < 0.05.

### Ethics approval and consent to participate

The study was conducted in accordance with the Guide for the Care and Use of Laboratory Animals of the National Institutes of Health and in accordance with ARRIVE guidelines . All animal studies reported in this study were approved by the institutional ethics committee of Ben-Gurion University of the Negev, Israel (Protocols IL4108-2016 and IL5908-2020D.

### Supplementary Information

Supplementary Information 1.

## Data Availability

All data generated or analyzed during this study are included in this publication. Additional information will be made available upon request (Sharon Etzion shar@bgu.ac.il).
