# Supplementary material for: Plekhm2 acts as an autophagy modulator in murine heart and cardiofibroblasts
Source: Sci Rep. 2024 Jun 28;14:14949. doi: 10.1038/s41598-024-65670-5 (PMC11213891; doi:10.1038/s41598-024-65670-5)
Supplement: Supplementary file 1 — Supplementary Information 1. [file 41598_2024_65670_MOESM1_ESM.pdf]

Plekhm2 acts as an autophagy modulator in murine heart and cardiofibroblasts

Sharon Etzion[1], Raneen Hijaze[1,2], Liad Segal[1,3], Sofia Pilcha[1], Dana Masil[1,2], Or Levi[1,3], Sigal Elyagon[1,3], Aviva Levitas[4], Yoram Etzion[1,3] and Ruti Parvari[2,5]

[1]Regenerative Medicine and Stem Cell (RMSC) Research Center, Ben-Gurion University of the Negev, Be'er-Sheva 84101, Israel

[2]Department of Microbiology, Immunology and Genetics, Faculty of Health Sciences, Ben-Gurion University of the Negev, Be'er-Sheva 84101, Israel

[3]Department of Physiology and Cell Biology, Faculty of Health Sciences, Ben-Gurion University of the Negev, Be'er-Sheva 84101, Israel

[4]Department of Pediatric Cardiology, Soroka University Medical Center, Ben-Gurion University of the Negev, Be'er-Sheva 84101, Israel

[5]National Institute for Biotechnology, Ben-Gurion University of the Negev, Be'er-Sheva 84101, Israel

Abbreviated title: Roles of Plekhm2 in the heart

Address for Correspondence: Sharon Etzion, Regenerative Medicine and Stem Cell (RMSC) Research Center, Ben-Gurion University of the Negev, P.O. Box 653, Be'er Sheva 84105, Israel. Email: shar@bgu.ac.il

Supplementary Materials and Methods

Generation of Plekhm2 floxed mice for Plekhm2 KO cell cultures

Plekhm2tm1a mice were mated with a mouse (JAX 009086) expressing the enzyme flippase ($FLP^{+/+}$). The flippase deletes the sequence between the FRT sites, thereby removing the LacZ+ Neo cassette and leaving only the LoxP sites flanking exon 8 (named Plekhm2tm1c = floxed Plekhm2 gene). Mice born from this pairing were interbred with WT mice ($FLP^{-/-}$) to obtain $Plekhm2^{floxed/floxed}$ /$FLP^{-/-}$ mice. Neonatal cells were isolated from $Plekhm2^{floxed/floxed}$/$FLP^{-/-}$ mouse hearts and incubated with adenovirus expressing the Cre-recombinase enzyme (as we previously described [1]), resulting in the deletion of exon 8 and Plekhm2 KO. Cells transfected with the control adenovirus express Plekhm2 protein normally.

Echocardiographic analysis

Two-dimensional images of the left ventricle were obtained in parasternal long- and short-axis views. Long- and short-axis M-mode images were taken at the mid-papillary muscle level with cursor penetration at the papillary muscle tip. The LV end-systolic diameter (LVDes) and LV end-diastolic diameter (LVDed) were evaluated from the long-axis M-mode trace. Calculations of LV fractional shortening (FS, %) were conducted using (LVDed − LVDes)/LVDed × 100. LV ejection fraction (LVEF) was calculated using planimetry as follows: EF = 100 × (LVD3ed − LVD3es/LVD3ed). The maximum duration of the echocardiographic procedure was 15 minutes.

Gene expression analysis by real-time fluorescent quantitative PCR

Total RNA was extracted from cells or hearts homogenized in TRI reagent using a Direct-zolTM RNA Mini-Prep kit (#R2050-1-50, Zymo Research) according to the manufacturer's instructions. RNA concentration and purity were measured using a NanoDrop1000 spectrophotometer (Thermo Fisher Scientific, MA, USA). cDNA synthesis was performed using random hexamers and TaqMan reverse transcription reagents according to the manufacturer's protocol (Quanta BioSciences, MA, USA). Gene expression was examined with reverse transcription-quantitative polymerase chain reaction (qPCR) and PerfeCTa SYBR Green FastMix (Quanta BioSciences) using a QuantStudio5 Real Time PCR System (Thermo Fisher Scientific). Reaction programs comprised pre-incubation of 50°C for 3 minutes and 95°C for 3 minutes followed by 40 cycles of 95°C for 10 seconds and 60°C for 45 seconds and a final dissociation stage. Relative gene expression was calculated by the efficiency $2^{-\Delta\Delta CT}$ method or as relative measurements ($2^{-\Delta CT}$) with the expression of the genes of interest (Table S5) normalized to that of the housekeeping gene GAPDH. Each sample was tested in triplicate.

Supplementary Figures

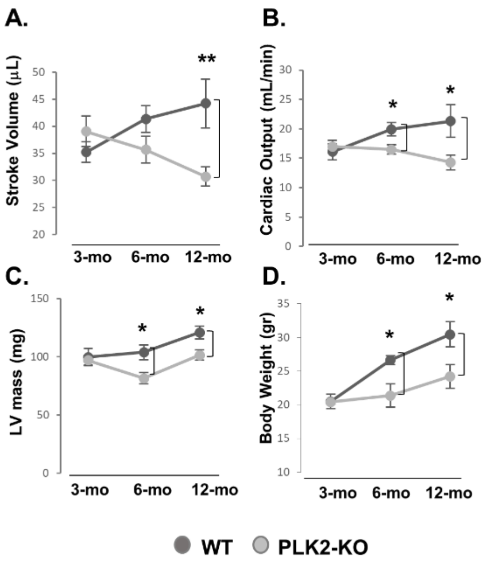

Fig. S1 Deterioration in the physiological parameters of PLK2-KO mice with aging. Echocardiography was conducted in 3-, 6-, and 12-month-old mice and several parameters were examined. **A.** Stroke volume (μL). **B.** Cardiac output (mL/min). **C.** LV mass (mg). **D.** Total body weight (g). Statistical analyses were conducted with GraphPad Prism. *p<0.05 and **p<0.01 for same-age PLK2-KO versus WT.

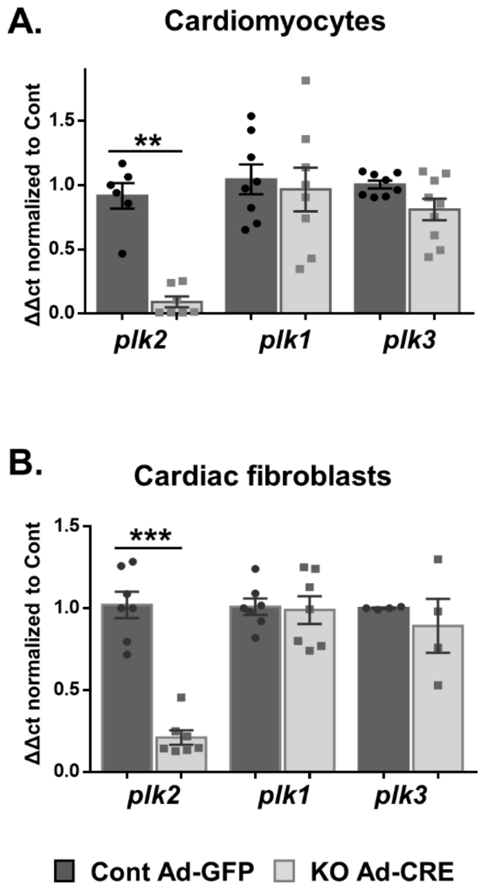

Fig. S2 Plekhm1 and Plekhm3 do not compensate for the absence of Plekhm2 in cardiac cells. Neonatal mice cardiomyocytes (**A**) and Neonatal mice cardiofibroblasts (**B**), obtained from mice with floxed Plekhm2 allele, were infected with adenovirus expressing Cre-recombinase (Ad-Cre) or GFP as control (Ad-GFP) for 5 days. Levels of *plk2*, *plk1*, and *plk3* mRNA were examined using RT-qPCR with specific primers. *Gpdh* served as an internal control. Data were normalized to control cells (Ad-GFP) and are displayed as mean±SEM (n=4–7 wells in each group from 2–3 different isolations). **p<0.01 and ***p<0.001 by Mann–Whitney nonparametric test.

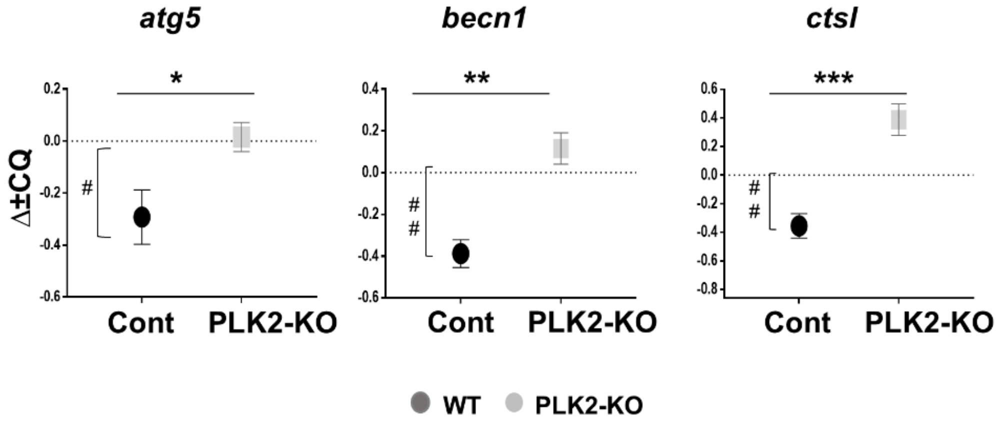

Fig. S3 Decrease in the expression of autophagy-related genes in control but not Plekhm2 KO-NMCFs. mRNA levels of *becn1*, *atg5,* and *ctsl* were evaluated in CQ-treated NMCFs and untreated cells. Calculation of the delta expression before and after CQ addition demonstrated that CQ significantly reduced *atg5*, *becn1*, and *ctsl* in control but not KO cells. Statistical analysis was conducted with a Wilcoxon signed-rank test and the column median was compared to a hypothetical value using GraphPad Prism software. $^{\#}p<0.05$ and $^{\#\#}p<0.01$ versus the hypothetical value of 0, and $^{*}p<0.05$ and $^{**}p<0.01$ for KO-NMCFs versus control following CQ (Mann–Whitney nonparametric test).

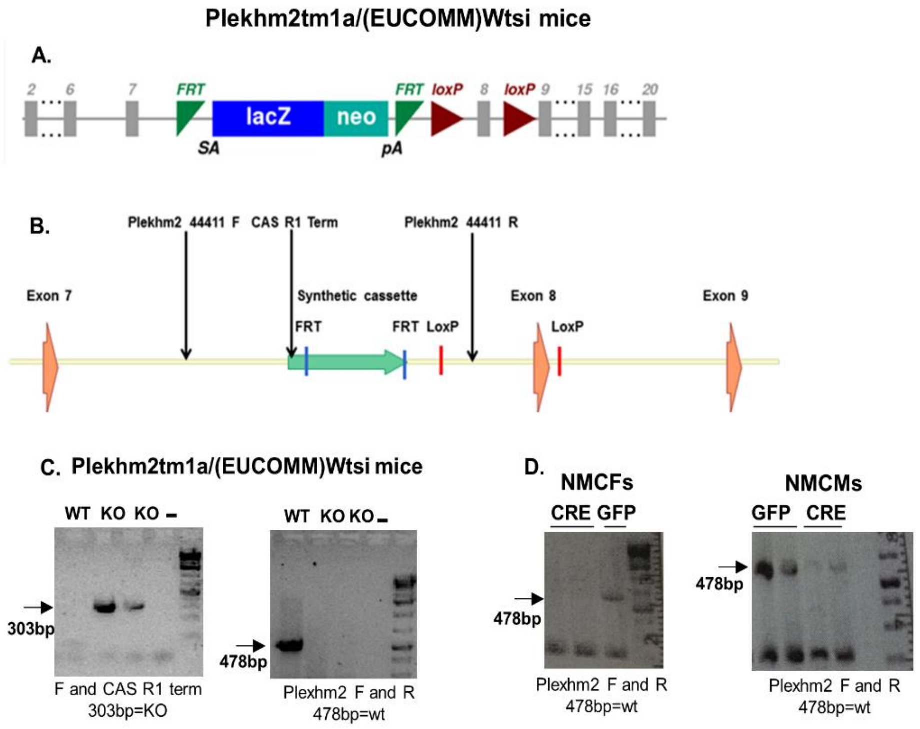

Fig. S4 Confirmation of Plekhm2 ablation in mice and primary murine cardiac cells. **A.** Schematics representing the Plekhm2tm1a(EUCOMM/Wtsi) mouse genotype. **B.** The positions of the primers used for genotyping. The primers Plekhm2 44411 F and Plekhm2 44411 R are on the normal genomic DNA flanking the cassette. The distance between them is too large to produce a PCR fragment when the cassette is inserted, representing KO mouse. When the lacZ-neo cassette is not present, the PCR product will comprise 478 bp, representing WT mouse. An additional primer, named CAS R1 Term, was used with Plekhm2 44411 F to verify the presence of the cassette. This will result in a 300-bp product **(C)**. When these mice are crossed with Flp mice, the sequence flanked by the FRT is removed. These Plekhm2$^{floxed/floxed}$/FLP$^{-/-}$ mice will further be used for neonatal cardiac cell isolation. **D.** For deletion of Plekhm2 from neonatal cells, we transfected the cells with Cre-recombinase enzyme. Because Plekhm2 44411 R primer is located between the LoxP sites, it will be removed by the Cre enzyme. Thus, no PCR product is expected for the KO cells. Plekhm2 deletion from NMCMs and NMCFs (CRE) versus control cells (GFP). The molecular size marker on the right side of the photographs is GeneRuler 1 kb Plus DNA Ladder of Thermo Fisher Scientific.

Supplementary Tables

Echocardiogram	3 months old		12 months old	
	**WT (n=6)**	**PLK2-KO (n=6)**	**WT (n=6)**	**PLK2-KO (n=6)**
Weight (g)	20.50 ± 1.06	20.40 ± 0.30	30.40 ± 1.88#	24.20 ± 1.74*
HR (bpm)	455.2 ± 29.7	432.5 ± 12.4	469.6 ± 28.1	461.8 ± 28.3
LVIDd (mm)	3.97 ± 0.12	3.74 ± 0.13	4.05 ± 0.15	3.66 ± 0.18
LVIDs (mm)	2.98 ± 0.11	2.66 ± 0.11	2.91 ± 0.17	2.65 ± 0.19
LVPWd (mm)	0.66 ± 0.04	0.77 ± 0.05	0.85 ± 0.04#	0.93 ± 0.12
LVPWs (mm)	0.94 ± 0.10	1.13 ± 0.08	1.15 ± 0.04	1.02 ± 0.10
EF (%)	48.8 ± 2.6	55.6 ± 2.8	54.4 ± 4.7	50.4 ± 2.3
FS (%)	24.4 ± 1.6	30.7 ± 2.6	28.1 ± 3.4	27.9 ± 3.0
LVVd (µL)	75.5 ± 4.9	73.9 ± 7.6	86.9 ± 8.8	63.2 ± 4.3*
LVVs (µL)	40.3 ± 3.1	45.3 ± 11.7	40.7 ± 6.9	32.0 ± 4.6
SV (µL)	35.2 ± 1.9	39.0 ± 2.9	44.2 ± 4.5	30.7 ± 1.8**,#
CO (mL/min)	16.1 ± 1.4	17.01 ± 1.02	21.3 ± 2.8	14.3 ± 1.3*
RWT (mm)	0.76 ± 0.04	0.77 ± 0.02	0.88 ± 0.04	0.87 ± 0.03
LV mass (mg)	99.7 ± 7.4	96.9 ± 4.1	120.8 ± 5.5#	101.4± 4.6*
**Gravimetric analysis**	**3 months old**		**12 months old**	
	**WT (n=10)**	**PLK2-KO (n=13)**	**WT (n=5)**	**PLK2-KO (n=8)**
BW (g)	20.8 ± 0. 8	20.1 ± 0.5	29.9 ± 1.9#	24.2 ± 1.3
HW (mg)	87.6 ± 3.6	91.4 ± 3.0	114.1 ± 3.4##	99.2 ± 2.8**
TL (mm)	17.5 ± 0.2	17.7 ± 0.1	18.7± 0.1##	17.9 ± 0.1**
HW/TL	5.29 ± 0.23	5.37 ± 0.23	6.10 ± 0.16#	5.52 ± 0.15**

Table S1 Basic characterization of PLK2-KO female mice. Basic physiological parameters and gravimetric measurements were examined in young (3-month-old) and aged (12-month-old) female PLK2-KO mice and their WT normal siblings. HR, heart rate; LVIDd, left ventricular internal diameter in diastole; LVIDs, left ventricular internal diameter in systole; LVPWd, left ventricular posterior wall in diastole; LVPWs, left ventricular posterior wall in systole; EF, ejection fraction; FS, fractional shortening; LVVd, left ventricular volume in diastole; LVVs, left ventricular volume in systole; SV, stroke volume; CO, cardiac output; RWT, relative wall thickness; BW, body weight; HW, heart weight; TL, tibia length. *$p<0.05$ and **$p<0.01$ for same-age KO versus WT; #$p<0.05$ and ##$p<0.01$ for 12-month-old WT versus 3-month-old WT.

**Echocardiographic measurements**	**WT (n=17)**	**PLK2-KO (n=20)**
Weight (g)	28.4 ± 0.66	25.8 ± 0.48*
HR (bpm)	476.5 ± 12.9	457.3 ± 8.88
LVIDd (mm)	3.91 ± 0.1	3.86 ± 0.08
LVIDs (mm)	2.71 ± 0.11	2.68 ± 0.09
LVPWd (mm)	0.93 ± 0.09	0.85 ± 0.03
LVPWs (mm)	1.28 ± 0.08	1.19 ± 0.04
EF (%)	58.7 ± 2.3	58.5 ± 1.96
FS (%)	30.9 ± 1.6	30.7 ± 1.32
SV (μL)	49.9 ± 1.44	43.2 ± 1.9*
CO (mL/min)	23.9 ± 1.77	21.3± 2.06
RWT (mm)	0.98 ± 0.05	0.90 ± 0.02
LV mass/BW	4.91 ± 0.23	4.46 ± 0.18

**Gravimetric analysis**	**WT (n=6)**	**PLK2-KO (n=10)**
BW (g)	28.9 ± 0.70	27.3 ± 0.46*
HW (mg)	128.0 ± 6.0	119.4 ± 4.71
TL (mm)	18.4 ± 0.21	18.3 ± 0.24
HW/TL	6.73 ± 0.19	6.67 ± 0.35

Table S2. Basic physiological parameters of 3-month-old male WT and PLK2-KO mice. Basic physiological parameters and gravimetric measurements were examined in young (3-month-old) male PLK2-KO and their WT normal sibling. HR, heart rate; LVIDd, left ventricular internal diameter in diastole; LVIDs, left ventricular internal diameter in systole; LVPWd, left ventricular posterior wall in diastole; LVPWs, left ventricular posterior wall in systole; EF, ejection fraction; FS, fractional shortening; LVVd, left ventricular volume in diastole; LVVs, left ventricular volume in systole; SV, stroke volume; CO, cardiac output; RWT, relative wall thickness; BW, body weight; HW, heart weight; TL, tibia length. *$p<0.05$ for KO versus WT.

6 months old	WT (n=6)	PLK2 KO (n=6)
Weight (g)	26.6 ± 0.65$^{\#}$	21.4 ± 1.74*
HR (bpm)	485.2 ± 12.1	457.8 ± 13.6
LVIDd (mm)	3.94 ± 0.12	3.52 ± 0.17
LVIDs (mm)	2.95 ± 0.19	2.53 ± 0.24
LVPWd (mm)	0.80 ± 0.04$^{\#}$	0.80 ± 0.03
LVPWs (mm)	1.11 ± 0.09	1.02 ± 0.08
EF (%)	49.9 ± 5.3	55.3 ± 5.7
FS (%)	25.5 ± 3.8	28.8 ± 3.6
LVVd (µL)	84.3 ± 42.9	70.3 ± 6.6
LVVs (µL)	42.9 ± 4.6	36.5 ± 7.2
SV (µL)	41.3 ± 2.9	35.6 ± 2.5
CO (mL/min)	19.9 ± 1.1	16.6 ± 0.8*
RWT (mm)	0.83 ± 0.03	0.80 ± 0.02
LV mass (mg)	103.9 ± 6.4$^{\#}$	81.6 ± 5.07$^{*,\#}$

Table S3. Deterioration in physiological parameters with aging. Basic physiological parameters and gravimetric measurements were examined in 6-month-old female PLK2-KO and their WT normal siblings. HR, heart rate; LVIDd, left ventricular internal diameter in diastole; LVIDs, left ventricular internal diameter in systole; LVPWd, left ventricular posterior wall in diastole; LVPWs, left ventricular posterior wall in systole; EF, ejection fraction; FS, fractional shortening; LVVd, left ventricular volume in diastole; LVVs, left ventricular volume in systole; SV, stroke volume; CO, cardiac output; RWT, relative wall thickness; BW, body weight; HW, heart weight; TL, tibia length. *$p<0.05$ for same-aged KO versus WT; $^{\#}p<0.05$ for 6-month-old WT or KO versus 3-month-old WT.

Gravimetric analysis	WT saline (n=7)	WT AngII (n=7)	PLK2-KO saline (n=7)	PLK2-KO AngII (n=8)
BW (g)	28.0 ± 0.8	24.8 ± 0.6*	25.8 ± 0.6	22.7± 0.7 *
HW (mg)	119.0 ± 5.6	167.7± 8.6***	118.7 ± 2.9	128.3 ± 3.8#
HW/BW	4. 3 ± 0.2	6.8 ± 0.3***	4.6 ± 0.1	5.7 ± 0.2**,#
**Echocardiography**	**WT saline (n=6)**	**WT AngII (n=7)**	**PLK2-KO saline (n=7)**	**PLK2-KO AngII (n=7)**
Weight (g)	29.9 ± 0.85	26.1 ± 0.56**	27.4 ± 0.57	24.3 ± 0.90*
HR (bpm)	483.7 ± 20.7	538.7 ± 12.4*	461.9 ± 25.1	555.9 ± 11.6*
LVIDd (mm)	4.23 ± 0.12	3.89 ± 0.17	3.82 ± 0.09	3.93 ± 0.15
LVIDs (mm)	3.04 ± 0.21	2.91 ± 0.24	2.53 ± 0.17	3.0 ± 0.24
LVPWd (mm)	0.66 ± 0.04	1.1 ± 0.08*	0.87 ± 0.049	0.86 ± 0.07#
LVPWs (mm)	1.09 ± 0.15	1.39 ± 0.11	1.25 ± 0.12	1.14 ± 0.11
EF (%)	54.7 ± 4.9	50.5 ± 5.4	62.9 ± 4.1	47.2 ± 5.9
FS (%)	28.6 ± 3.2	25.9 ± 3.5	34.1 ± 3.2	23.9 ± 3.5
SV (µL)	49.1 ± 1.9	52.8 ± 23.7	39.4 ± 2.5	31.7± 2.0###
CO (mL/min)	23.8 ± 1.8	28.4± 1.9	20.4 ± 2.0	17.4 ± 1.09###
RWT (mm)	0.93 ± 0.03	1.1 ± 0.04*	0.98 ± 0.05	1.0 ± 0.05
LV mass (mg)	139.2 ± 6.1	183.0 ± 13.5*	137.9 ± 9.1	137.8± 8.6#

Table S4 Physiological and gravimetric analysis Gravimetric analysis and echocardiographic measurements following 2 weeks of angiotensin-II infusion were examined in PLK2-KO mice and their WT normal siblings. BW, body weight; HW, heart weight; TL, tibia length; HR, heart rate; LVIDd, left ventricular internal diameter in diastole; LVIDs, left ventricular internal diameter in systole; LVPWd, left ventricular posterior wall in diastole; LVPWs, left ventricular posterior wall in systole; EF, ejection fraction; FS, fractional shortening; LVVd, left ventricular volume in diastole; LVVs, left ventricular volume in systole; SV, stroke volume; CO, cardiac output; RWT, relative wall thickness. *p<0.05 and **p<0.01 for WT versus WT-Ang or KO versus KO AngII; #p<0.05, ##p<0.01, and ###p<0.001 for KO AngII versus WT AngII.

Primer sequence (5'-3')-R	Primer sequence (5'-3')-F
Plekhm2r TCGTCCAGCTTGGTCTTTTT	Plekhm2f GCGTCATAACCCCTTCAATG
Plekhm1r AGCTTGGGTCGTACAAAGGA	Plekhm1f ACTTGGTGGGAGTCTGGATG
Plekhm3r GGCAGGGGACAGACTTTTCT	Plekhm3f CTGTAACAACGGCGAGATCC
mActa1r CCACCGATCCACACTGAGTA	Acta1f AAGTGCGACATCGACATCAG
mActa2r CACCAGGGCTGTGCTGTCTT	Acta2f AGCCAGTCGCTGTCAGGAA
Nppar AATGTGACCAAGCTGCGTGA	Nppaf GCTGCAACAGCTTCCGGTA
Nppbr TGGTCCTTCAAGAGCTGTCTC	Nppbf AGGTGCTGTCCCAGATGATT
Myh7r TCCACGATGGCGATGTTCT	Myh7f CCTCCAGAGTCTGCTGAAGGA
TGFb1r TGGTTGTAGAGGGCAAGGAC	TGFb1f TTGCTTCAGCTCCACAGAGA
LCBr CGCCGTCTGATTATCTTGATG	LCBf CCACCAAGATCCCAGTGATTATAG
P62r TGGGAGAGGGACTCAATCAG	P62f ACAGATGCCAGAATCGGAAG
BCln1r ATCTTGCCTTTCTCCACGTC	BCln1f TTTGACCATGCAATGGTAGC
Col1a1r GACGTGCTTCTTTTCCTTGG	Col1a1f TGACTGGAAGAGCGGAGAGT
Col3a1r GTCACCATTTCTCCCAGGAA	Col3a1f CAATATGCCCACAGCCTTCT
Col1a2r TGGGACCATCAACACCATC	Col1a2f TGCTCAGCTTTGTGGATACG
ATG5r CGGAACAGCTTCTGGATGA	ATG5f CAACCGGAAACTCATGGAAT
Mctsl1r CATAGCCATAGCCCACCAAC	Mctsl1f TCTGTTGCTATGGACGCAAG
KIF5Br TTCTACAATCCCAAGGAATAGAGG	KIF5Bf GGAGGCAAGCAGTCGTAAAC
KLC1r CCATGCTCTCAGGGTCATTT	KLC1f AGCTGCAGAGACATTGGAAGA
Arl8Br CTTCTCCGGGATTTTGAGTG	Arl8Bf GCCTTGGATGAGAAACAGCTA
Rab7ar CTGGCCTGGATGAGAAACTC	Rab7af CTGACCAAGGAGGTGATGGT
Gapdhr CCAATACGGCCAAATCCGT	Gapdhr TCTTGTGCAGTGCCAGCCT

Table S5. Primer sequences used in this study

Supplementary References

1. Segal, L. *et al.* Dock10 Regulates Cardiac Function under Neurohormonal Stress. *International journal of molecular sciences* **23** (2022). http://doi.org/10.3390/ijms23179616
